# Physiological Responses and Absorption Removal Effects of Three Floating Macrophytes to Polystyrene Nanoplastics Pollution

**DOI:** 10.3390/toxics14090802

**Published:** 2026-09-10

**Authors:** Yuanmiao Chen, Jianhui Xue, Jianfeng Hua, Zhidong Zhou

**Affiliations:** 1 Jiangsu Provincial Key Laboratory for Plant Taxonomy, Resource Conservation and Utilization, Institute of Botany, Jiangsu Province and Chinese Academy of Sciences (Nanjing Botanical Garden Mem. Sun Yat-Sen), Nanjing 210014, China; 2 Co-Innovation Center for Sustainable Forestry in Southern China, College of Ecology and Environment, Nanjing Forestry University, Nanjing 210037, China

**Keywords:** free-floating macrophytes, polystyrene nanoplastics (PS-NPs), physiological and biochemical characteristics, phytoremediation

## Abstract

Microplastics exert toxic effects on aquatic plants, thereby impacting ecosystem functions and services. Currently, research on the effects of nanoplastics on aquatic plants has mainly focused on algae, while floating macrophytes have received less attention. To investigate the physiological responses and uptake and accumulation effects of floating macrophytes to polystyrene nanoplastics (PS-NPs), we exposed *Limnobium laevigatum*, *Pistia stratiotes* and *Eichhornia crassipes* to 0.1, 1 and 10 mg·L^−1^ of PS-NPs (20/100 nm) for 45 days. The results showed that, in general, with increasing concentration and exposure time, the three plant species exhibited a decrease in chlorophyll content, a decline in root activity, an increase in cell membrane permeability and an up-regulation of antioxidant enzyme activities. Meanwhile, the contents of proline, soluble sugars and soluble proteins showed dynamic changes. These findings indicate that under PS-NPs stress, the plants suffered structural damage and they alleviated oxidative injury and maintained osmotic balance by actively accumulating osmoregulatory substances and up-regulating antioxidant enzyme activities. Furthermore, the effects of uptake and accumulation PS-NPs differed among species. At low concentration levels, *Limnobium laevigatum* exhibits higher uptake and accumulation potential, while at high concentrations, *Pistia stratiotes* demonstrates greater uptake and accumulation potential.

## 1. Introduction

Microplastics (MPs) are defined as plastic particles with a diameter of less than 5 mm [1], with those below 100 nm referred to as nanoplastics (NPs) [2]. They are primarily formed through the photochemical degradation, physical abrasion, and biodegradation of larger plastic debris in the environment [3]. In recent years, global plastic production has continued to grow, leading to an increasing abundance of nanoplastics in aquatic environments. Lakes, rivers and oceans are now widely contaminated [4,5,6]. NPs have even been detected in remote areas such as the polar regions and the deep sea [7,8,9]. NPs have a small size and a large specific surface area, allowing them to enter, accumulate and transfer within organisms, leading to toxic effects [10,11]. Furthermore, NPs may undergo biomagnification along food chains and food webs [12,13], resulting in enhanced toxicity. Aquatic plants are the primary components of aquatic ecosystems, making them more susceptible to NPs pollution. NPs can enter the roots, stems and leaves of aquatic plants, accumulating in intercellular spaces [14,15,16]. This process affects plant growth, cellular structure, photosynthesis and osmotic balance [17,18,19,20]. Generally, nanoplastics stress triggers the accumulation of reactive oxygen species (ROS) in plants [21]. This leads to lipid peroxidation and increased cell membrane permeability, damaging organelles such as the nucleus, mitochondria and chloroplasts [22,23]. Consequently, these effects result in reduced chlorophyll content, decreased photosynthetic rates and inhibited growth [24]. However, the toxic effects of NPs on plants are regulated by multiple factors, including species, plastic type, concentration, shape and particle size [25,26,27,28]. This can lead to contrasting outcomes—such as promotion, inhibition or no effect—on the same indicator. For instance, one study [14] found that stress from 1 µm PS-MPs significantly inhibited the growth of *Phragmites australis* and *Spirodela polyrrhiza*, but had no significant effect on *Vallisneria natans*. Conversely, another study reported that exposure to 5 µm and 50 µm PS-MPs led to an increase in the biomass of *Hydrilla verticillata* and *Elodea nuttallii* [29]. Therefore, research conclusions derived from a limited number of plant species are difficult to generalize to other plant groups. Current toxicological research on nanoplastics in aquatic plants remains significantly imbalanced. While a large number of studies focus on algae, insufficient attention has been paid to large aquatic plants, particularly floating macrophytes, and their physiological responses remain unclear. Moreover, algae as simple non-vascular plants, differ significantly from large vascular plants in their physiological structure, reproductive modes and metabolic pathways [30,31]. Therefore, toxicity data based solely on algae are insufficient to comprehensively assess the risks of nanoplastics to the entire aquatic plant community. To systematically evaluate the impact of nanoplastics on aquatic plants, it is necessary to strengthen research on large floating macrophytes.

Current research on remediation technologies for microplastic pollution in aquatic environments has focused on exploiting the physicochemical properties of microplastics to remove them from water bodies through mechanisms such as adsorption [32], filtration [33], flocculation and sedimentation [34], catalytic degradation [35], and bioaccumulation [15]. Although these physicochemical techniques achieve high removal efficiencies for microplastics, they require substantial energy input and carry the risk of secondary pollution. Bioremediation, particularly phytoremediation, offers lower costs and reduced environmental impact, presenting considerable application prospects [36,37]. Phytoremediation has been widely used to treat eutrophication and heavy metal pollution and has recently been applied to nanoplastics contaminated waters [38,39]. For example, microalgae facilitate the removal of microplastics through the formation of hetero-aggregates via the secretion of extracellular polymeric substances and by enhancing the adsorption of microplastics onto solid surfaces [40], large aquatic plants remove microplastics from water through absorption and adsorption entrapment [41,42]. Yuan et al. [15] compared the absorption and accumulation capacities of submerged, emergent and floating plants for PS-MPs. They found that floating plants exhibited higher absorption potential, suggesting a unique advantage in remediating microplastic pollution. Yin et al. [42] reported that *Eichhornia crassipes* primarily removes microplastics from water through root adsorption. Within 48 h, the removal efficiencies of *Eichhornia crassipes* for 0.5, 1 and 2 µm polystyrene particles reached 55.3%, 69.1% and 68.8%, respectively. *L. laevigatum*, *P. stratiotes* and *E. crassipes* are three floating species characterized by well-developed root systems and rapid reproduction [43,44,45]. While they are effective in treating eutrophication and heavy metal pollution [46,47,48], their capacity to absorb nanoplastics has been overlooked. Determining the differences in their nanoplastics absorption capabilities could help expand the selection of plants for managing co-existing pollution from nutrients, heavy metals and nanoplastics.

Therefore, this study was designed to investigate the physiological responses and absorption and accumulation characteristics of *L. laevigatum*, *P. stratiotes*, and *E. crassipes* under PS-NPs stress. This study contributes to a deeper understanding of the impacts of nanoplastics on the physiological and biochemical reactions of floating macrophytes. It provides a scientific basis for selecting appropriate plant species for the phytoremediation of water contaminated by nanoplastics and offers new insights into the synergistic treatment of eutrophication, heavy metal pollution and nanoplastics contamination. Furthermore, this research holds both theoretical and practical significance for the expansion and application of phytoremediation technologies.

## 2. Materials and Methods

### 2.1. Materials

In this study, spherical red fluorescent polystyrene nanoplastics and europium-doped polystyrene nanoplastics, with nominal particle sizes of 20 nm and 100 nm, were purchased from Shanghai Huge Biotechnology Co., Ltd., Shanghai, China. The morphology of the PS-NPs particles was examined by transmission electron microscopy (JEM-2100 UHR, JEOL, Tokyo, Japan). The particle sizes of the 20 and 100 nm red fluorescent and europium-doped PS-NPs were 29.18 ± 4.73, 97.65 ± 5.05, 31.21 ± 4.42 and 100.79 ± 7.56 nm, respectively. Their actual morphology and size distribution are shown in the Figure S1. The Zeta potentials of the NPs in 10% Hoagland nutrient solution were measured using a nanoparticle size and Zeta potential analyzer (Malvern Zetasizer Pro, Malvern Panalytical, Malvern, UK). The Zeta potentials of the 20 nm and 100 nm red fluorescent and europium-doped PS-NPs were −18.15 ± 0.87, −24.45 ± 1.01, −18.68 ± 1.04 and −25.77 ± 0.65 mV, respectively.

The emission spectra of these PS-NPs were measured using a steady-state and time-resolved fluorescence spectrometer (FluoroMax-4, HORIBA, Palaiseau, France) at the specified excitation wavelengths. These spectra are presented in Figure S2. *Limnobium laevigatum*, *Pistia stratiotes* and *Eichhornia crassipes* were obtained from a horticultural base in Suqian, Jiangsu Province, China.

### 2.2. Experimental Design

Current investigations of microplastic pollution in water bodies predominantly employ abundance as the metric, while studies using mass concentration as the standard are relatively scarce. In these limited studies, the reported concentrations of microplastics in freshwater are mostly below 5 mg·L^−1^ [49,50,51,52]. Nevertheless, Previous studies have often focused on microplastic concentrations far exceeding those found at realistic environmental levels, lacking environmental relevance [24,42,53,54]. This may lead to an overestimation of their impacts on aquatic plants. Consequently, this study established a concentration gradient of to investigate the physiological responses and uptake and accumulation potential of three floating macrophytes under realistic pollution levels.

The experiment was conducted from April to May 2025 in the Institute of Botany, Jiangsu Province and Chinese Academy of Sciences (The daily average air temperature ranged from 14 to 29 °C during the experimental period). *L. laevigatum*, *P. stratiotes* and *E. crassipes* were thoroughly rinsed and pre-cultured in 10% Hoagland nutrient solution under natural sunlight for 10 days. The experimental design included five treatments with three replicates each: small particle size and low concentration (D1C1, 20 nm 0.1 mg·L^−1^ PS-NPs), small particle size and medium concentration (D1C2, 20 nm 1 mg·L^−1^ PS-NPs), small particle size and high concentration (D1C3, 20 nm 10 mg·L^−1^ PS-NPs), large particle size and high concentration (D2C3, 100 nm 10 mg·L^−1^ PS-NPs) and a control (CK, no PS-NPs). Spherical and europium-doped PS-NPs were added to 2 L of 10% Hoagland solution according to the design and homogenized via ultrasonication in 2.5 L polypropylene rectangular containers. After pre-culturing, healthy plants of uniform size were selected. Initial biomass was maintained at approximately 75 g per treatment group, with the same number of individuals per species (Plants were cultivated in a monoculture system, with each container housing only one species. The initial planting densities were 10 individuals of *L. laevigatum*, 5 individuals of *P. stratiotes*, and 4 individuals of *E. crassipes* per container, respectively). They were then transferred to the aforementioned culture containers and cultivated for 45 days. The nutrient solution was replenished to its initial volume (2 L) twice weekly. Environmental conditions during the experiment remained consistent with those of the pre-culture period.

### 2.3. Sample Collection

Fresh plant samples were collected on days 3, 10, 24 and 45 of the culture periods. Samples treated with spherical red fluorescent PS-NPs were used for the determination of physiological indicators and the tracing of PS-NPs within plant tissues. Samples treated with europium doped PS-NPs were used to quantify the internal concentration of accumulated polystyrene nanoplastics.

### 2.4. Measurement of Physiological Indicators and Quantification of PS-NPs

#### 2.4.1. Physiological Indicators and Tracing of PS-NPs

The methods for measuring chlorophyll (Chl) content, root activity, cell membrane permeability, soluble sugar (SS) concentration, soluble protein (SP) content, malondialdehyde (MDA) content, proline content (Pro) and antioxidant enzyme activities are shown in Text S1 [55]. The method for tracing PS-NPs within plant tissues is described in Text S2 [56].

#### 2.4.2. Quantification of PS-NPs in Plant Tissues

The concentration of PS-NPs in plant tissues was quantified using the europium (Eu) tracer method [15]. Initially, a series of standard solutions were prepared by diluting europium-doped PS-NPs, which were then digested and analyzed using Inductively Coupled Plasma Mass Spectrometry (ICP-MS; NEXION 300, PE, Waltham, MA, USA) to establish a standard curve. Subsequently, the roots and leaves of the harvested plants were separated, oven-dried, and ground into a fine powder. A 0.25 g sample of the powder was placed into a digestion tube with 5 mL of HNO_3_ and 1 mL of H_2_O_2_. The mixture was digested at 180 °C for 3 h using a graphite digestion system (SH230N, Hanon, Jinan, China) until complete mineralization. After digestion, the samples were diluted with ultrapure water and transferred to centrifuge tubes. The concentration of Eu ions in the samples was measured via ICP-MS, and the background values from the control group were subtracted. The mass concentration of europium-doped PS-NPs in the tissues was then calculated based on the standard curve. Finally, the total PS-NPs concentration in the whole plant was determined according to the dry weight ratio of roots to leaves for each of the three species. The standard curve is shown in Figure S3.

### 2.5. Statistical Analysis

Data processing was performed using Excel 2019. Statistical analyses were performed using SPSS 29.0 One-way and two-way analyses of variance (ANOVA) were conducted. For data with homogeneity of variance, post-hoc comparisons were performed using Fisher’s Least Significant Difference (LSD) test. In cases of heterogeneity of variance, Tamhane’s T2 test was employed. All figures were generated using Origin 2021.

To assess the adequacy of the sample size, post-hoc power analysis was performed based on the F values obtained from two-way ANOVA. The analysis was conducted with a significance level of α = 0.05, five NPs treatments, four time points, and three replicates per treatment combination (total sample size N = 60). Statistical power was calculated using the pwr package in R 4.0.3, with power values ≥ 0.8 considered indicative of adequate sample size. Detailed results of the power analysis are provided in Table S1.

## 3. Results

### 3.1. Changes in Chlorophyll Content

**Figure 1:**
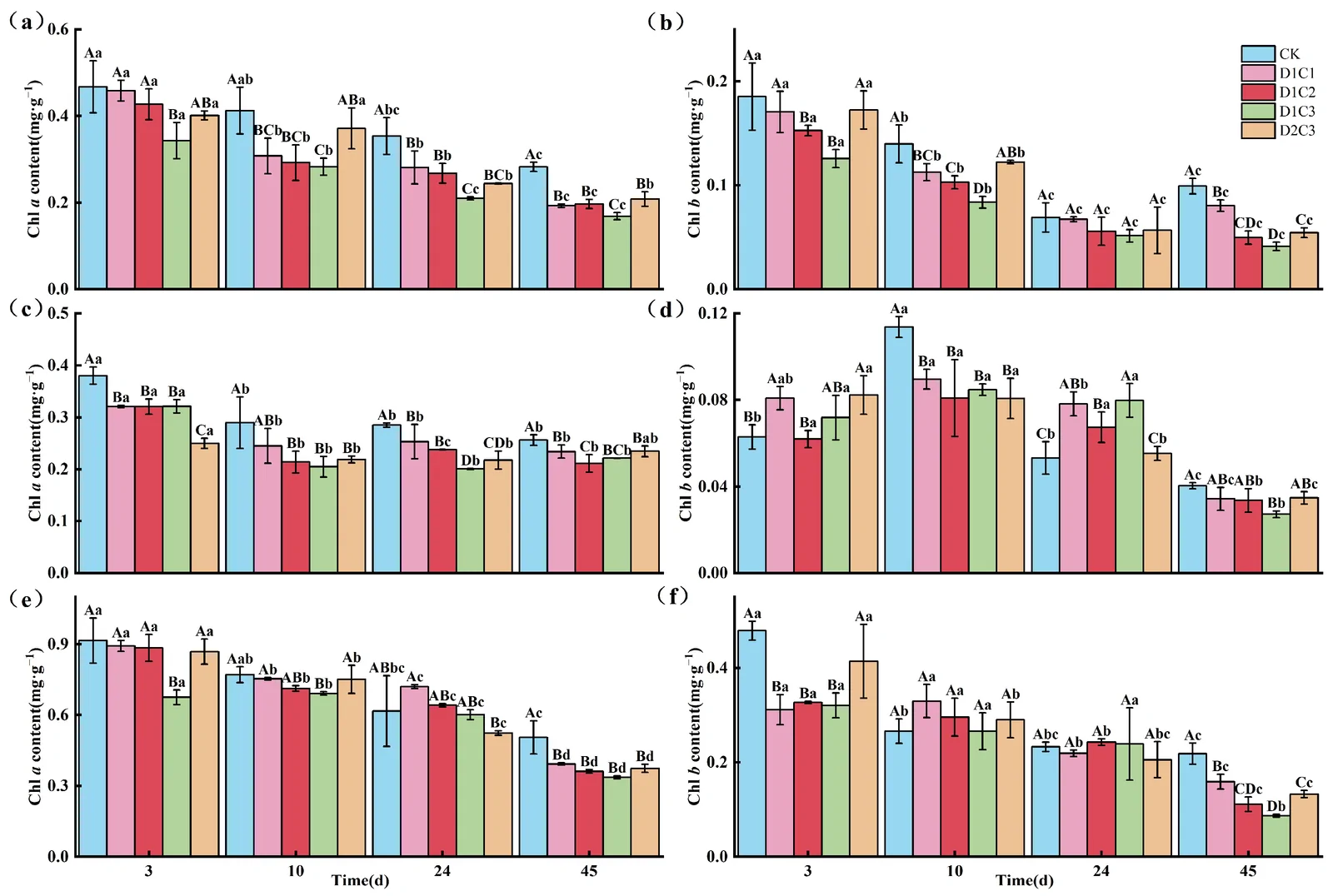
Effect of PS-NPs on Chl a content and Chl b content in three plant species. (**a**,**b**) *L. laevigatum*, (**c**,**d**) *P. stratiotes*, (**e**,**f**) *E. crassipes*. Different capital letters denote significant differences among treatments at the same sampling time (*p* < 0.05); different lowercase letters denote significant differences over time within the same treatment (*p* < 0.05).

PS-NPs treatments had a significant impact on the chlorophyll *a* content of all three plant species (*p* < 0.05) (Figure 1a,c,e; Table S2). For *L. laevigatum*, chlorophyll *a* level in all experimental groups were lower than those in the CK, with the D1C3 treatment showing the most substantial decrease. Similarly, the chlorophyll *a* content in *P. stratiotes* remained lower than the CK across all PS-NPs treatments. On days 3, 10 and 24, the most pronounced inhibitory effects on *P. stratiotes* were observed in the D2C3 and D1C3 groups; however, by day 45, the D1C2 treatment exhibited the greatest impact. For *E. crassipes*, chlorophyll *a* content was higher than the CK only under the D1C1 and D1C2 treatments on day 24; at all other time points, PS-NPs stress resulted in lower levels compared to the CK. Overall, the D1C3 and D2C3 treatments exerted the strongest influence on the chlorophyll *a* content of the three species.

Nanoplastics, exposure time and their interaction had significant effects on the chlorophyll *b* content of the three plant species (Figure 1b,d,f; Table S2). Exposure to PS-NPs resulted in a consistently lower chlorophyll *b* content in *L. laevigatum* compared to the CK throughout the study period. The D1C3 treatment exerted the most substantial impact, resulting in the greatest reduction in chlorophyll *b* content throughout the experimental period. The chlorophyll b content in *P. stratiotes* fluctuated markedly over time, exhibiting a dynamic “increase-decrease-increase-decrease” pattern. Similarly, the chlorophyll *b* content of *E. crassipes* showed a degree of fluctuation under PS-NPs stress. Compared to the CK, chlorophyll *b* levels in all *E. crassipes* treatment groups decreased on days 3 and 45. On day 10, only the D1C3 group showed a decrease, while other groups increased; by day 24, levels in the D1C2 and D1C3 groups increased, whereas other treatments showed a decline. These results indicate that the effect of PS-NPs on the chlorophyll *b* content of *L. laevigatum* is clearly correlated with nanoplastics concentration and particle size, whereas such correlations are weaker for the other two species.

**Figure 2:**
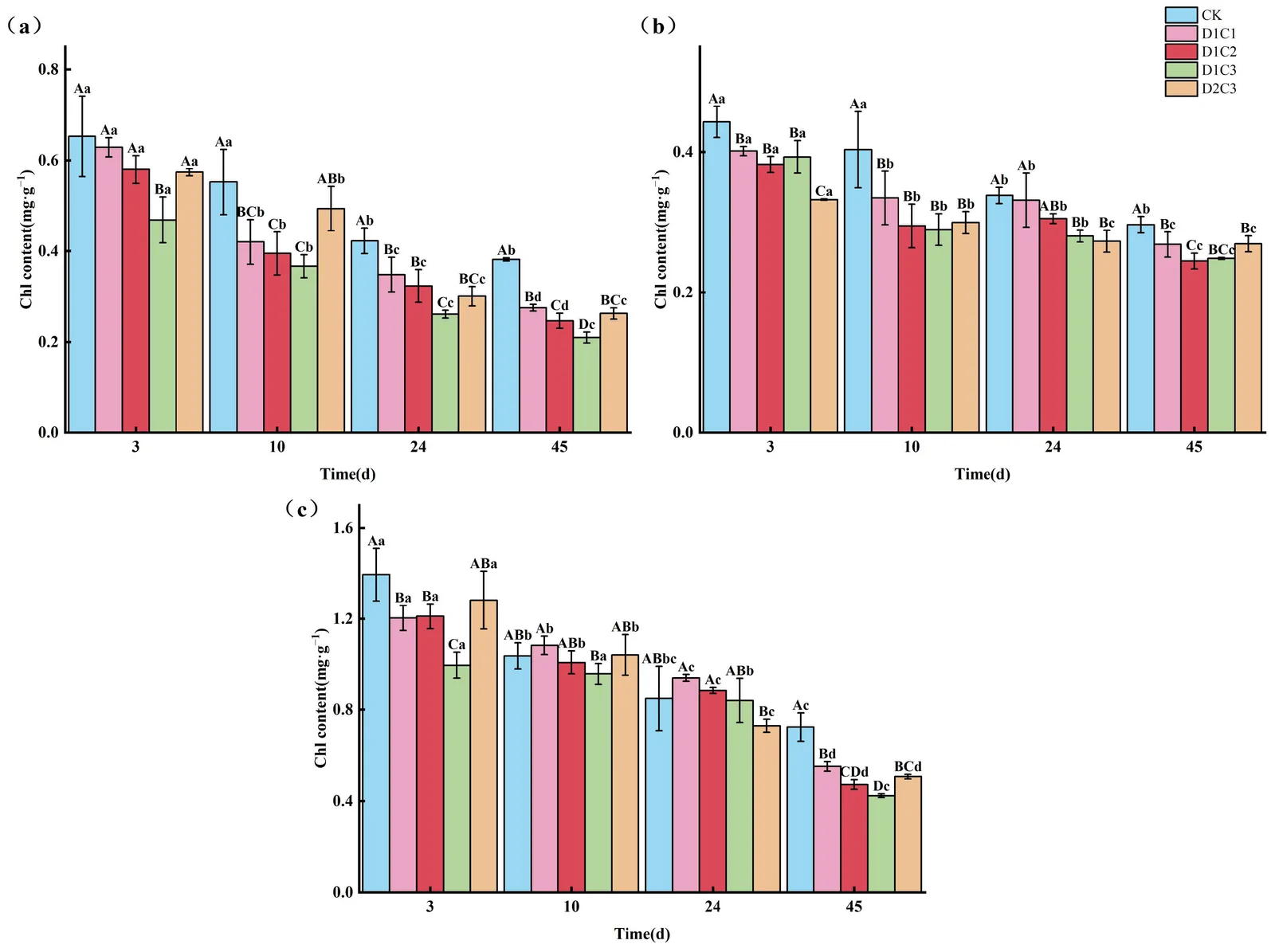
Effect of PS-NPs on Chl content in three plant species. (**a**) *L. laevigatum*, (**b**) *P. stratiotes*, (**c**) *E. crassipes*. Different capital letters denote significant differences among treatments at the same sampling time (*p* < 0.05); different lowercase letters denote significant differences over time within the same treatment (*p* < 0.05).

PS-NPs stress significantly affected the total chlorophyll content of all three plant species (*p* < 0.05) (Figure 2). For *L. laevigatum*, the total chlorophyll content was lower than the CK across all treatments, with the D1C3 group showing the most substantial reduction. Similarly, the total chlorophyll content of *P. stratiotes* remained below the CK in all treatment groups; specifically, on days 3, 10 and 24, the D1C3 and D2C3 treatments exhibited the greatest decreases compared to the CK, whereas on day 45, the D1C2 treatment showed the most significant decline. For *E. crassipes*, the D1C1 treatment increased the total chlorophyll content on days 10 and 24 compared to the CK, while the D1C3 treatment consistently reduced the total chlorophyll content at all sampling time points.

### 3.2. Changes in Root Activity and Cell Membrane Permeability

**Figure 3:**
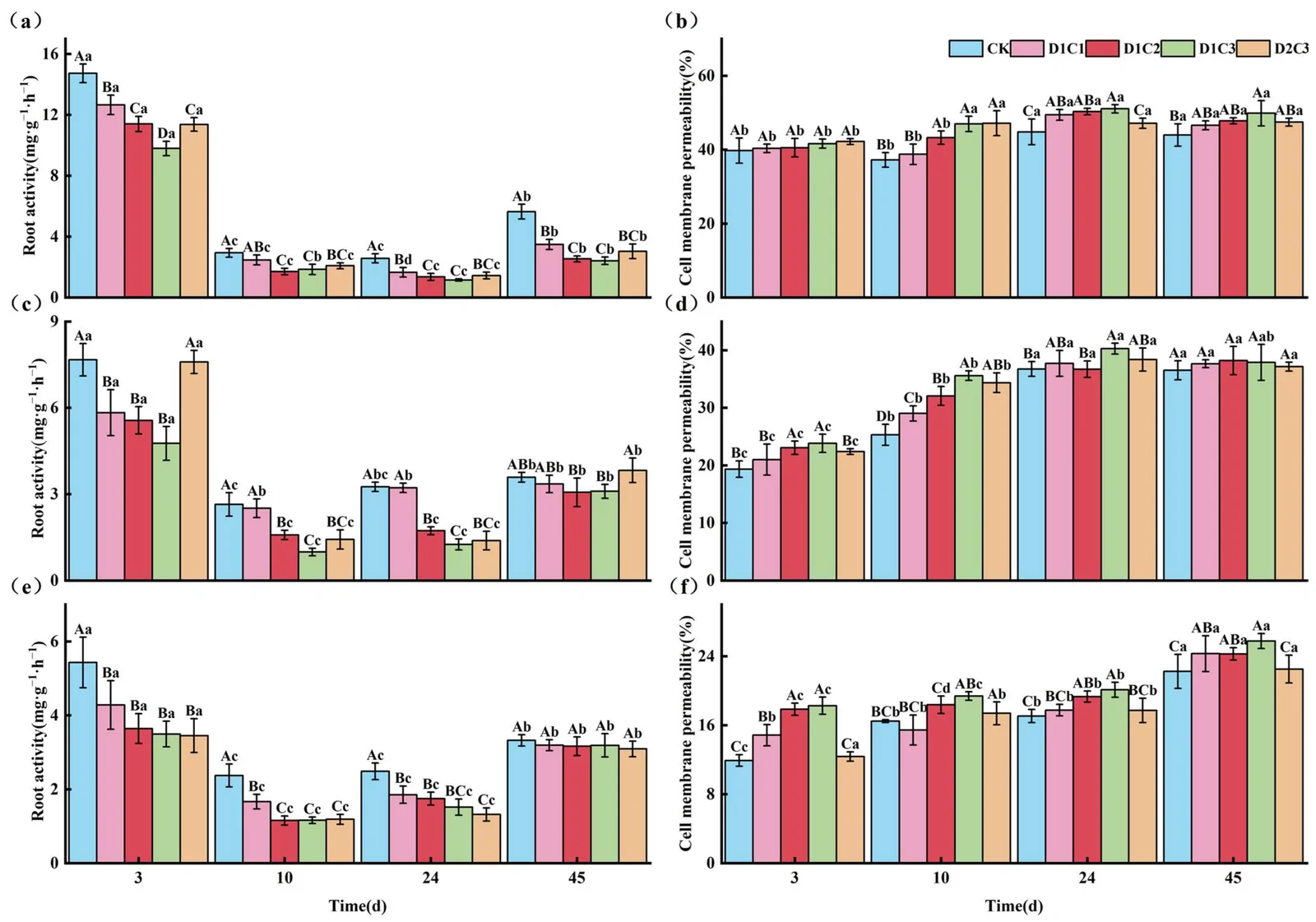
Effect of PS-NPs on root activity and cell membrane permeability in three plant species. (**a**,**b**) *L. laevigatum*, (**c**,**d**) *P. stratiotes*, (**e**,**f**) *E. crassipes*. Different capital letters denote significant differences among treatments at the same sampling time (*p* < 0.05); different lowercase letters denote significant differences over time within the same treatment (*p* < 0.05).

PS-NPs treatments, exposure time and their interaction significantly affected the root activity of all three plant species (*p* < 0.05) (Figure 3a,c,e; Table S2). For *L. laevigatum*, root activity remained consistently lower than the CK across all treatments throughout the experimental period. On days 3, 24 and 45, the most pronounced decrease in root activity was observed in the D1C3 group, while on day 10, the D1C2 treatment showed the greatest reduction compared to the CK. The root activity of *P. stratiotes* was lower than the CK at all sampling time points, except for the D2C3 group on day 45, which was slightly higher than the CK. On days 3, 10 and 24, the D1C3 treatment resulted in the greatest decrease compared to the CK, while the D1C2 group showed the most significant decline on day 45. Similar to *L. laevigatum*, root activity in all *E. crassipes* treatment groups was lower than that of the control. Specifically, the D2C3 treatment caused the most substantial reduction compared to the CK on days 3, 24 and 45, whereas the D1C2 group showed the greatest decrease on day 10. These results indicate that small-sized PS-NPs at medium and high concentrations (D1C2 and D1C3) exerted a greater inhibitory effect on plant root activity than the low concentration, small sized (D1C1) and high concentration large, sized (D2C3) treatments.

PS-NPs, exposure time and their interaction significantly affected the cell membrane permeability of all three plant species (*p* < 0.05) (Figure 3b,d,f; Table S2). Throughout the culture period, the degree of impact on cell membrane permeability increased with rising PS-NPs concentrations, with the D2C3 and D1C3 treatments exerting the greatest effects. For *L. laevigatum*, cell membrane permeability in the experimental groups remained consistently higher than that of the CK. For *P. stratiotes*, permeability was higher than the CK across all treatments and time points, except for a slight decrease under the D1C2 treatment on day 24. Similarly, the cell membrane permeability of *E. crassipes* was lower than the CK only under the D1C1 treatment on day 10, while it remained higher than the CK in all other treatments.

### 3.3. Changes in Soluble Sugar, Soluble Protein and Proline Contents

**Figure 4:**
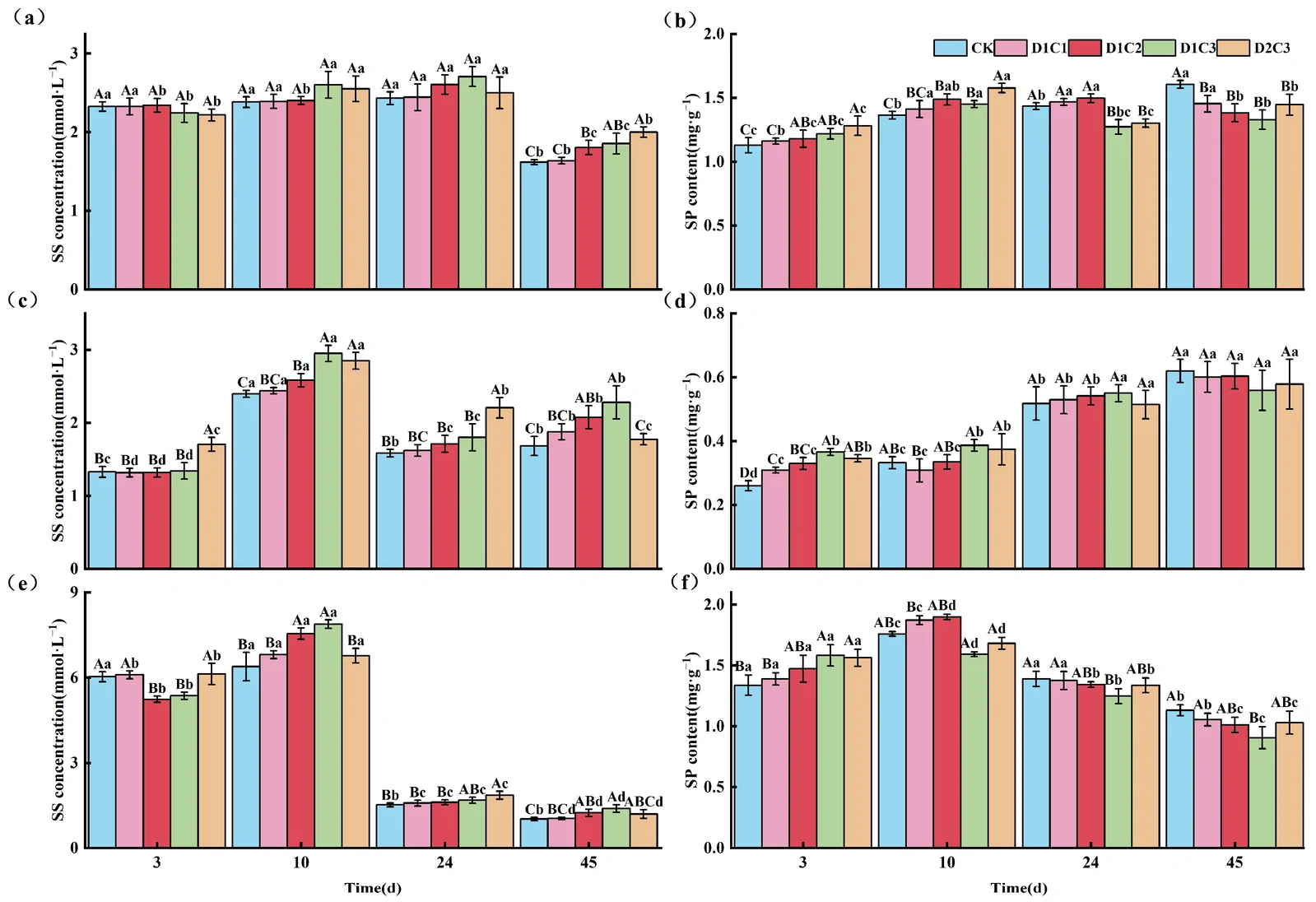
Effect of PS-NPs on SS and SP in three plant species. (**a**,**b**) *L. laevigatum*, (**c**,**d**) *P. stratiotes*, (**e**,**f**) *E. crassipes*. Different capital letters denote significant differences among treatments at the same sampling time (*p* < 0.05); different lowercase letters denote significant differences over time within the same treatment (*p* < 0.05).

Overall, the impact of PS-NPs on the SS concentration of the three plant species increased with rising nanoplastics concentrations and exhibited a trend of an initial increase followed by a subsequent decrease over the culture period (Figure 4a,c,e). PS-NPs stress, exposure time and their interaction had significant effect on the SS concentrations of *L. laevigatum*, *P. stratiotes and E. crassipes* (*p* < 0.05) (Table S2). On day 3, the SS concentrations of *L. laevigatum* under the D1C3 and D2C3 treatments were lower than those of the CK; however, as the culture period progressed, these concentrations gradually recovered and eventually exceeded the CK levels. The D1C3 and D2C3 treatments exerted the greatest influence on *P. stratiotes*, with SS concentrations being markedly higher than those of the CK. Similar to *L. laevigatum*, the SS concentrations of *E. crassipes* under the D1C2 and D1C3 treatments were lower than those of the CK on day 3, but surpassed the CK levels in the later stages of the experiment, while SS concentrations in all other treatment groups remained consistently higher than those of the CK.

PS-NPs treatments had a significant effect on the SP content of *L. laevigatum*, but no significant effect on that of *P. stratiotes* and *E. crassipes* (Table S2). Compared with the CK, the SP content in all three species exhibited a trend of an initial increase followed by a subsequent decrease over the experimental period (Figure 4b,d,f). Specifically, SP levels in the treatment groups were higher than the control in the early stages but fell below the control in the later stages. For *L. laevigatum*, SP content was generally higher than the CK on days 3 and 10, with the D2C3 group showing the most substantial increase. Starting from day 24, SP levels in the D1C3 and D2C3 groups dropped below the CK; by day 45, SP content in all treatment groups was lower than the CK, with D1C3 exhibiting the greatest reduction. The variation in SP content for *P. stratiotes* followed a similar pattern. On days 3, 10 and 24, SP levels in the experimental groups were generally higher than the control, with the highest concentration observed in the D1C3 group. By day 45, SP content in all treatments declined, and the D1C3 group showed the most significant decrease compared to the CK. *E. crassipes* showed a comparable response under PS-NPs stress. On day 3, all experimental groups had higher SP content than the CK, with D1C3 showing the largest increase. Subsequently, except for the D1C1 and D1C2 groups which remained briefly higher than the CK on day 10, SP levels in all other treatment groups were lower than the CK on days 10, 24 and 45.

**Figure 5:**
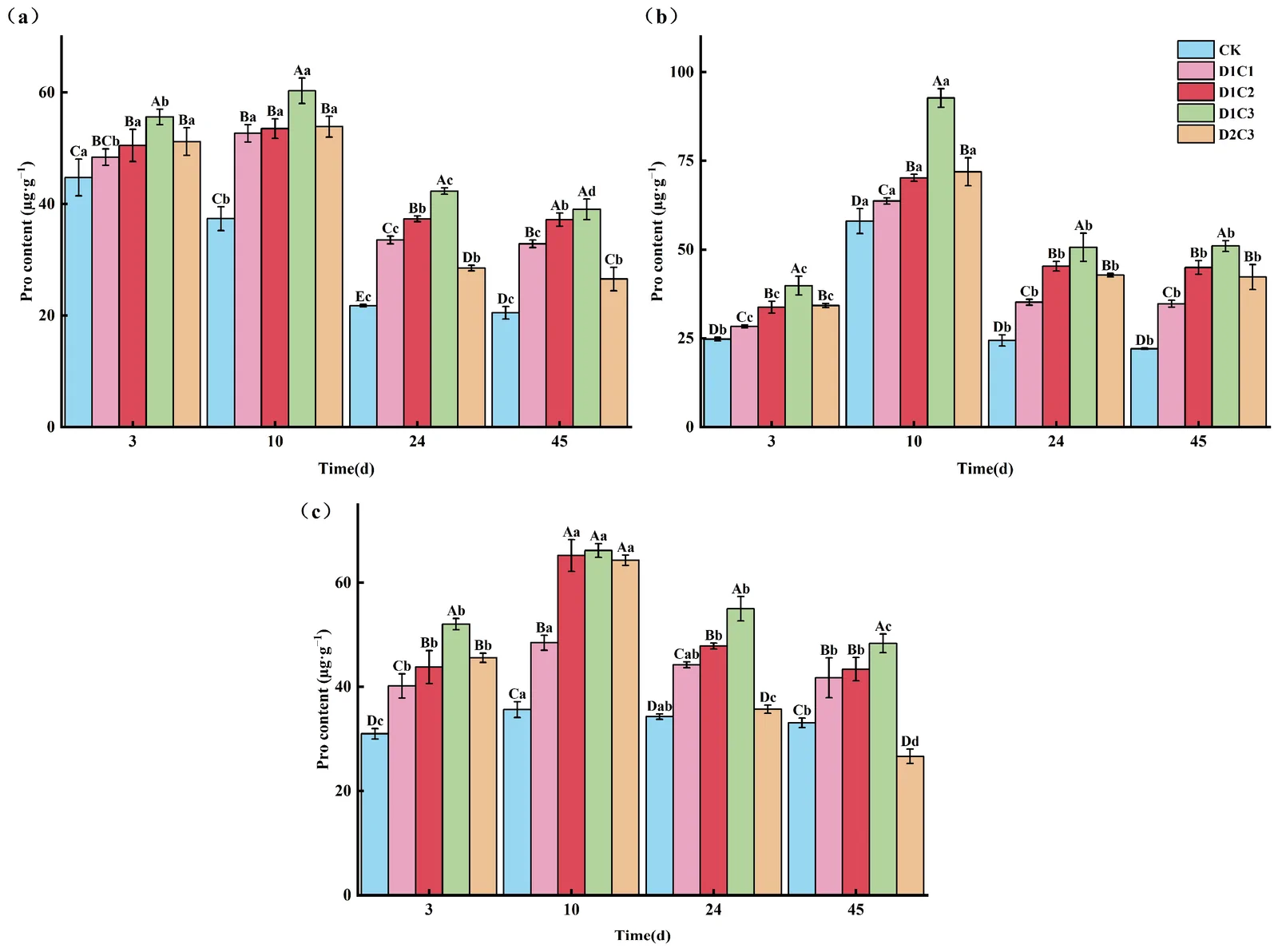
Effect of PS-NPs on Pro content in three plant species. (**a**) *L. laevigatum*, (**b**) *P. stratiotes*, (**c**) *E. crassipes*. Different capital letters denote significant differences among treatments at the same sampling time (*p* < 0.05); different lowercase letters denote significant differences over time within the same treatment (*p* < 0.05).

PS-NPs treatments, exposure time and their interaction significantly affected the Pro content of all three plant species (*p* < 0.05), with levels in the experimental groups generally remaining higher than those in the control (Figure 5; Table S2). Among the treatments, D1C3 exerted the most substantial impact on plant Pro content. Compared to the CK, the D1C3 treatment resulted in the greatest increase in Pro levels across all four sampling time points, which was significantly higher than the increase observed in the large sized, high concentration treatment (D2C3). This may be attributed to the fact that the toxic effects of NPs are closely related to their surface area or particle number. At the same mass concentration, the number of 20 nm PS-NPs is far higher than that of 100 nm PS-NPs, resulting in 20 nm PS-NPs exerting significantly greater physiological effects on plants than 100 nm PS-NPs [57].

### 3.4. Changes in Malondialdehyde Content and Antioxidant Enzyme Activities

**Figure 6:**
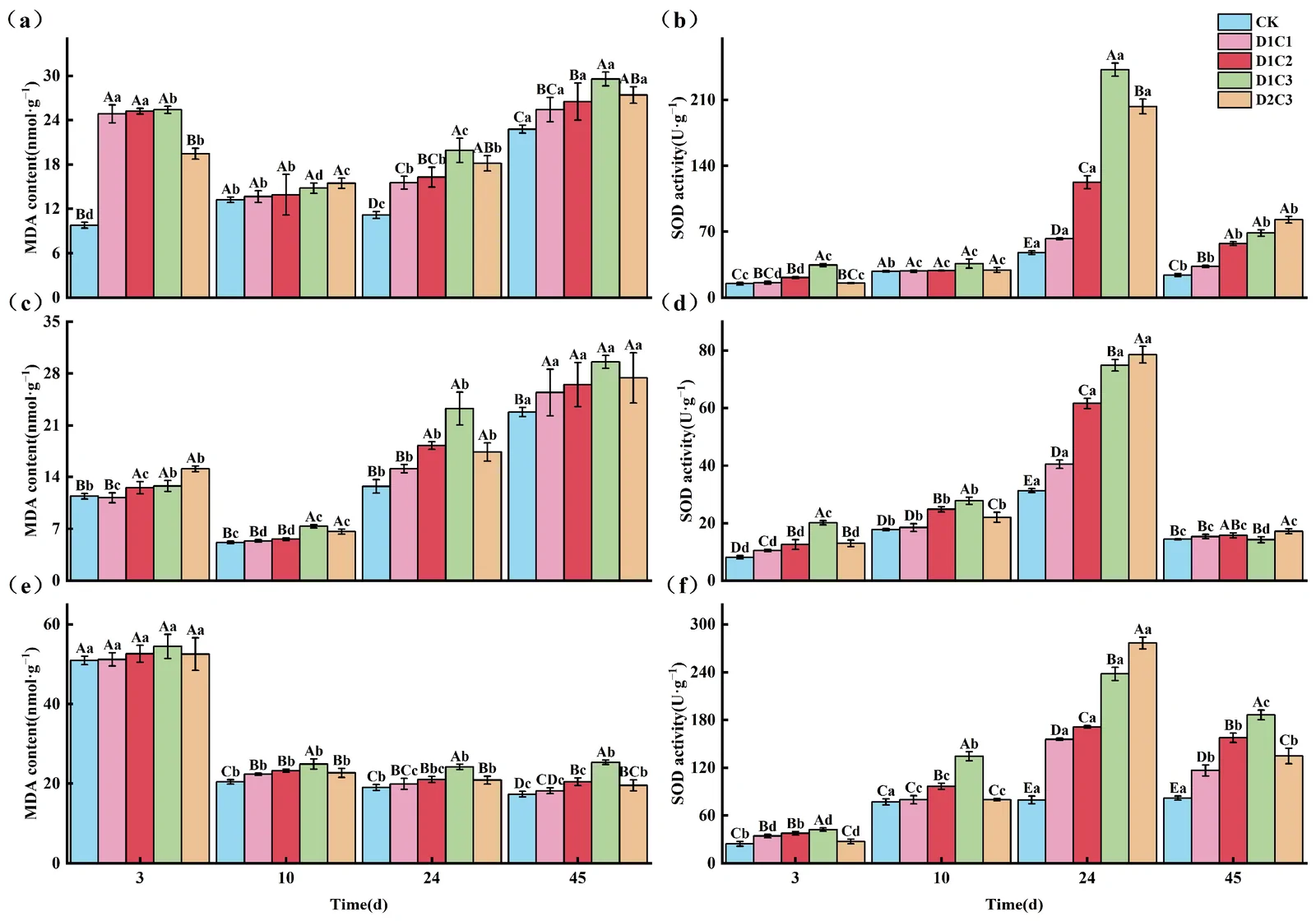
Effect of PS-NPs on MDA content and SOD activity in three plant species. (**a**,**b**) *L. laevigatum*, (**c**,**d**) *P. stratiotes*, (**e**,**f**) *E. crassipes.* Different capital letters denote significant differences among treatments at the same sampling time (*p* < 0.05); different lowercase letters denote significant differences over time within the same treatment (*p* < 0.05).

PS-NPs treatments significantly affected the MDA content of all three plant species (*p* < 0.05), leading to an overall increase in concentration (Figure 6a,c,e). This effect was positively correlated with NPs concentration, as higher concentrations resulted in greater increases in MDA content. Across the four sampling periods, peak MDA levels for all three species occurred in the D1C3 and D2C3 groups, indicating that high concentrations of PS-NPs exerted the most significant impact on MDA accumulation.

PS-NPs stress, exposure time and their interaction significantly affected the leaf superoxide dismutase (SOD), peroxidase (POD) and catalase (CAT) activities of all three plant species (*p* < 0.05). SOD activity in the experimental groups was generally higher than in the control and increased with rising NPs concentrations (Figure 6b,d,f). Higher concentrations of NPs exerted a greater influence on SOD activity, with peak values for *L. laevigatum*, *P. stratiotes*, and *E. crassipes* recorded in the D1C3 or D2C3 treatments. Specifically, the SOD activity of *L. laevigatum* was highest under the D1C3 treatment. For *P. stratiotes*, D1C3 induced the highest activity during the early stages, whereas D2C3 became the most effective treatment by day 45. A similar pattern was observed for *E. crassipes*, where the highest activity occurred in the D1C3 group on days 3 and 10, shifting to the D2C3 group in the later stages. Temporally, SOD activity in all three species peaked on day 24 and subsequently declined, exhibiting an “initial increase followed by a decrease” trend over time.

**Figure 7:**
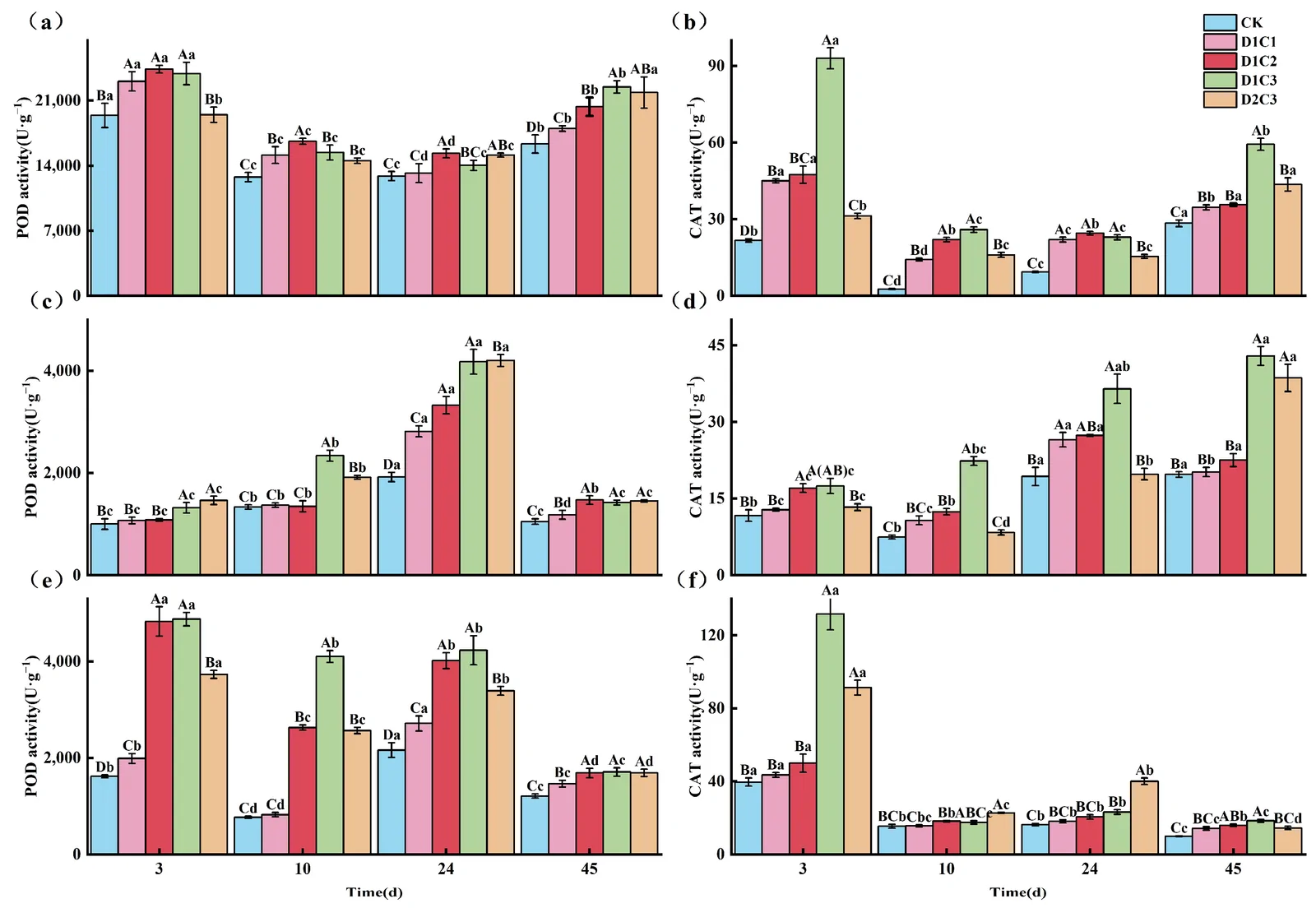
Effects of PS-NPs on POD and CAT activities in three plant species. (**a**,**b**) *L. laevigatum*, (**c**,**d**) *P. stratiotes*, (**e**,**f**) *E. crassipes*. Different capital letters denote significant differences among treatments at the same sampling time (*p* < 0.05); different lowercase letters denote significant differences over time within the same treatment (*p* < 0.05).

PS-NPs treatments increased the POD activity in all three plant species, with levels in the experimental groups consistently exceeding those of the CK. Furthermore, the variation in POD activity was correlated with nanoplastics concentration (Figure 7a,c,e). For *L. laevigatum*, POD activity exhibited a “decrease followed by an increase” trend over time. In *P. stratiotes*, POD activity generally enhanced with increasing PS-NPs concentrations and followed an “initial increase followed by a decrease” temporal trend. Unlike *L. laevigatum*, the high-concentration groups showed the highest activity during the early stages of treatment, shifting to the medium-concentration groups in the later stages. For *E. crassipes*, POD activity increased with concentration at all four sampling points; the small-sized, high-concentration PS-NPs group (D1C3) consistently maintained the highest activity, which was generally higher than that of the large-sized treatment groups.

PS-NPs treatments similarly increased the CAT activity of the three plant species, with all experimental groups exhibiting higher activity than the control (Figure 7b,d,f). The responses of CAT activity to PS-NPs were comparable among the three species, showing a positive correlation with nanoplastics concentration and generally peaking under high-concentration treatments. At the same concentration level, CAT activity in the small-sized PS-NPs groups was typically higher than that in the large-sized groups.

### 3.5. Accumulation of PS-NPs in Plant Tissues

**Figure 8:**
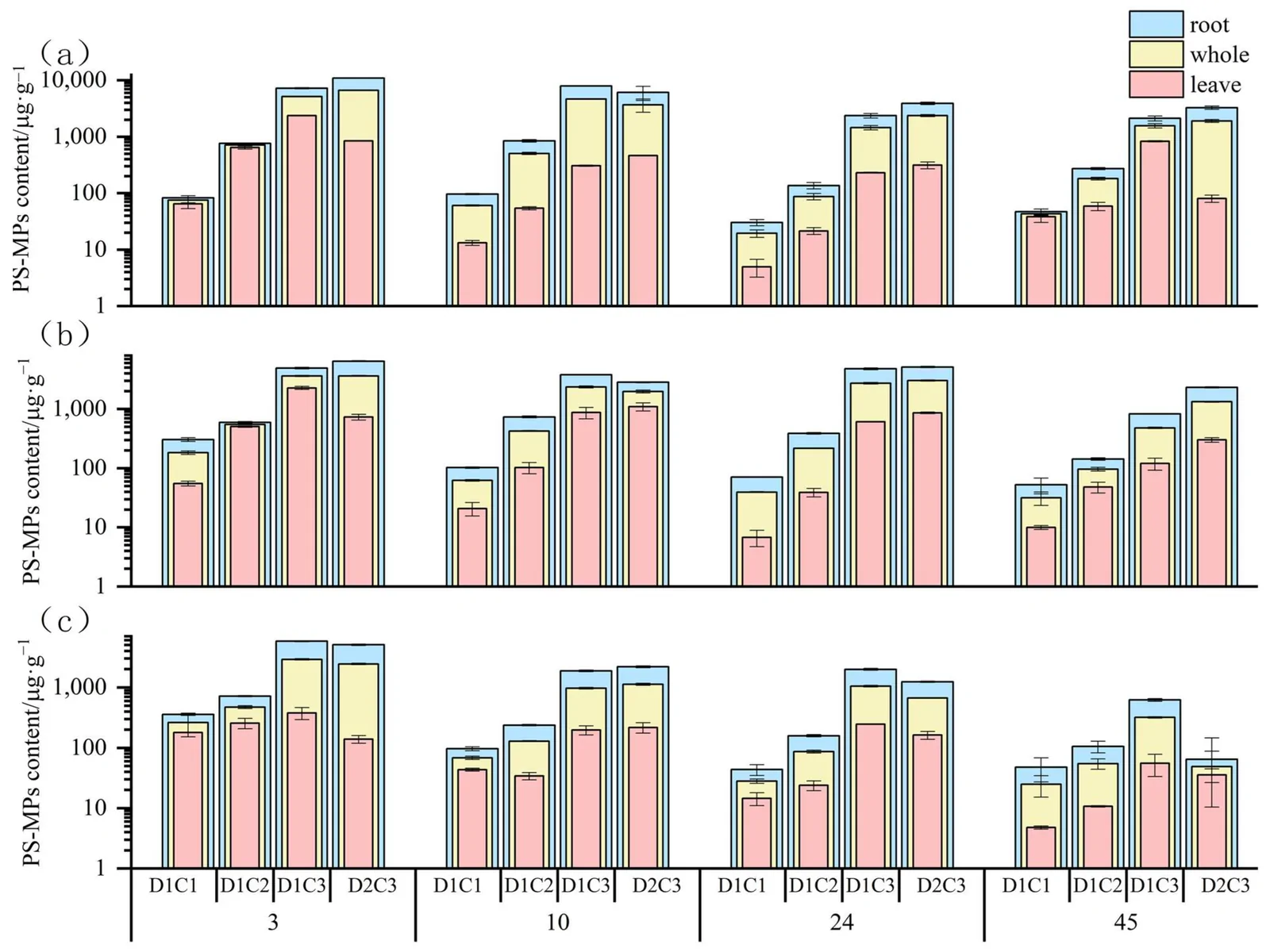
PS-NPs content in three plant species. (**a**) *L. laevigatum*, (**b**) *P. stratiotes*, (**c**) *E. crassipes.*

**Figure 9:**
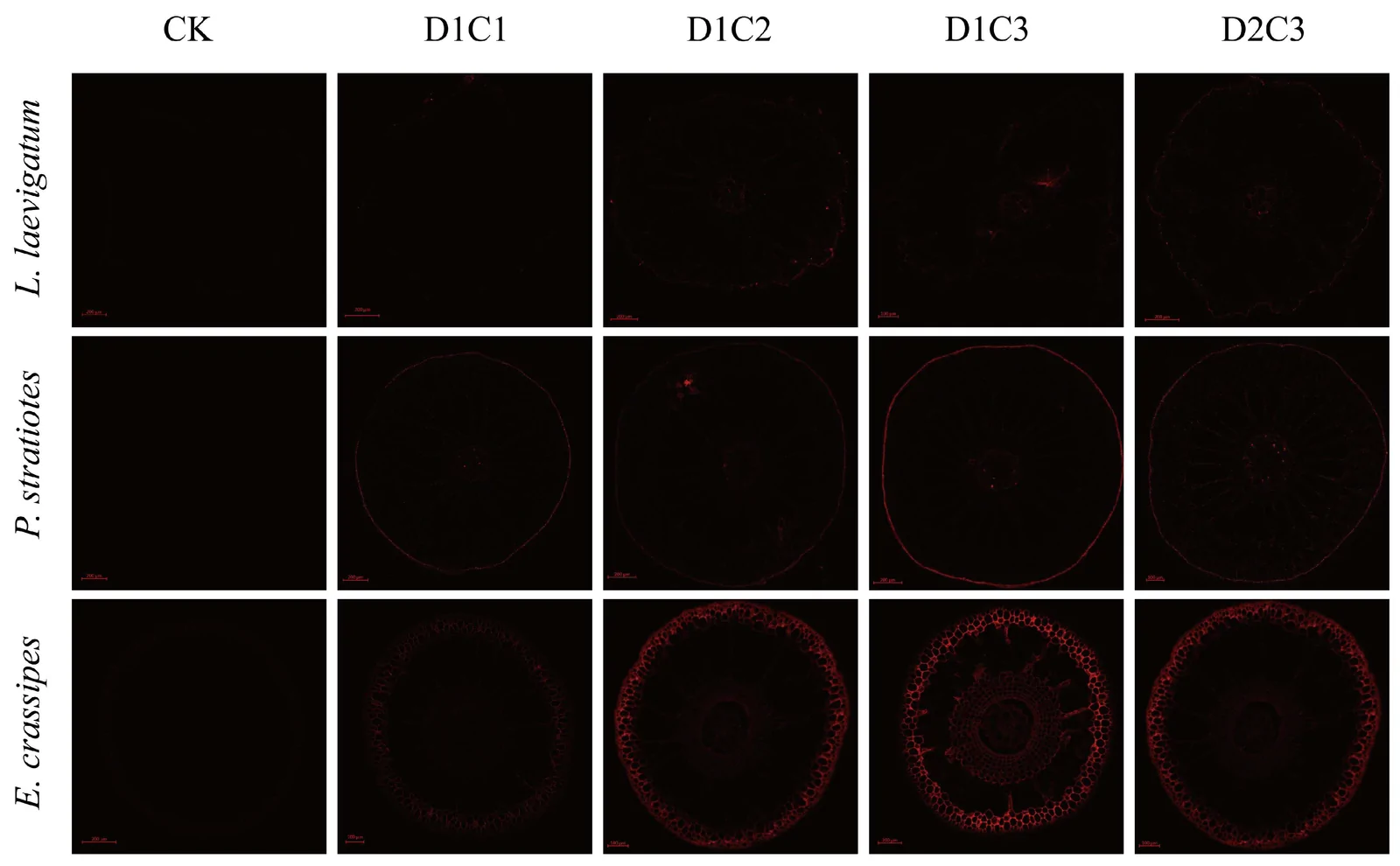
Laser confocal microscopy image of three plant roots.

Under PS-NPs treatments, the concentration of nanoplastics in the roots, leaves and whole plants of the three species exhibited a decreasing trend as the culture period progressed, with accumulation in the roots being generally higher than in the leaves (Figure 8). Higher exposure concentrations in the experimental groups led to higher nanoplastics content in the plant tissues. At the same concentration level, the accumulation of 20 nm PS-NPs in the leaves was higher than that of 100 nm PS-NPs, whereas the opposite trend was observed in the roots. As shown in Figure 9, by day 3, the average PS-NPs concentration ranges for *L. laevigatum* in the roots, leaves and whole plant were 82.66–10,934.26, 64.72–2357.32 and 74.97–6610.34 µg·g^−1^ (DW), respectively. For *P. stratiotes*, the corresponding concentration ranges were 305.27–6399.51, 55.29–2266.4 and 182.62–3617.82 µg·g^−1^ (DW). For *E. crassipes*, the ranges were 356.34–5765.45, 139.29–377.74 and 261.4–2887.66 µg·g^−1^ (DW). On day 3, under the D1C1 and D1C2 treatments, the whole plant PS-NPs concentrations followed the order: *L. laevigatum* > *P. stratiotes* > *E. crassipes*. However, under the D1C3 and D2C3 treatments, the order shifted to: *P. stratiotes* > *L. laevigatum* > *E. crassipes*.

## 4. Discussion

### 4.1. Effects of PS-NPs on Plant Chlorophyll Content

Chlorophyll plays a core role in light absorption, and changes in its content reflect the impact of NPs stress on plant photosynthesis. Overall, PS-NPs reduced the contents of chlorophyll *a* and total chlorophyll, with the magnitude of the decrease being concentration-dependent. Specifically, chlorophyll levels under high-concentration NPs stress were significantly lower than those under low-concentration stress. These findings are consistent with previous studies on *Hydrilla verticillata* [58], *Vallisneria natans* [59] and lettuce [60]. The reduction may be attributed to the destruction of chloroplast structures and the inhibition of chlorophyll synthesis following the internal accumulation of NPs [61,62]. Interestingly, *E. crassipes* exhibited an increase in chlorophyll *a* and total chlorophyll content under low concentration PS-NPs stress (0.1 and 1 mg·L^−1^). This aligns with the results reported by Zhang et al. in strawberries [63], potentially indicating an adaptive compensatory response triggered by mild stimulation from low concentration NPs. Furthermore, the impact of PS-NPs on the chlorophyll *b* content of *P. stratiotes* and *E. crassipes* showed a dynamic “inhibition, promotion, inhibition” pattern over time. This phenomenon might be related to the up-regulation of antioxidant enzyme activities during the mid-term of stress, which scavenges excess ROS and creates temporarily favorable conditions for chlorophyll synthesis.

### 4.2. Effects of PS-NPs on Root Activity and Cell Membrane Permeability

Root activity and cell membrane permeability are critical physiological indicators reflecting structural damage in plants under NPs stress [64,65]. In this study, PS-NPs stress led to a significant decrease in the root activity of the three tested species, with the most pronounced reduction observed under high-concentration treatments. This trend aligns with the findings of Yu et al. regarding *Salvinia cucullata* [66]. The decline in root activity can be attributed to two main factors. On one hand, nanoplastics particles adsorb and aggregate on the root surface [67,68] or enter the stele through intercellular spaces [53,69]. The resulting accumulation within the roots interferes with the uptake of water and nutrients, thereby weakening physiological activity. On the other hand, nanoplastics stress may trigger the massive accumulation of ROS in the roots. Excess ROS can attack cell membranes and induce lipid peroxidation, leading to increased membrane permeability and structural damage [61]. This disruption of normal metabolism ultimately results in the observed decline in root activity [17,23].

Changes in cell membrane permeability reflect the impact of nanoplastics on plants at the cellular level [65]. In this study, PS-NPs stress increased the leaf cell membrane permeability of the three plant species, with the magnitude of the increase positively correlated with nanoplastics concentration. This result is consistent with previous findings in algae [70,71] and wheat cells [61], further confirming the universality of nanoplastics-induced membrane damage. The mechanism likely involves nanoplastics stress stimulating the intracellular accumulation of ROS, which attack the membrane system [54,72]. This triggers a lipid peroxidation chain reaction that disrupts the phospholipid bilayer, leading to the loss of selective permeability, electrolyte leakage and a subsequent increase in relative electric conductivity [73].

### 4.3. Effects of PS-NPs on Plant Osmoregulatory Substances

SS, SP and Pro are key osmoregulatory substances that enable plants to maintain osmotic balance and resist external stress [74]. Overall, the SS content of the three tested species increased under PS-NPs stress, which is consistent with the findings of Zhou et al. [75] in *Oryza sativa* L. The SP content of the three species exhibited distinct dynamic characteristics, increasing in the early stages of stress and decreasing in the later stages. This result differs from the continuous upward trend observed by Li et al. [76] in a 10-day experiment with *Chlamydomonas reinhardtii* under PS-NPs stress. This discrepancy may stem from differences in experimental duration. The longer experimental period in this study allowed for the observation of a transition from active adaptation to structural damage and passive energy depletion under chronic stress, whereas shorter experiments may only capture the initial defense activation phase. The trend of an initial increase followed by a subsequent decrease in SS and SP content suggests that plants actively accumulate osmoregulatory substances in the early stages of stress to maintain osmotic balance. However, as the stress period extends, the decline in chlorophyll content and the resulting inhibition of photosynthesis lead to an insufficient supply of energy and synthetic substrates [77]. Coupled with continuous cell structure damage and intensified metabolic consumption, this ultimately results in the decline of SS and SP levels.

In addition to its role as an osmoregulant for maintaining cellular osmotic balance, Pro also functions as an antioxidant to mitigate oxidative damage [78]. In this study, the Pro content in all three plant species increased under PS-NPs stress, with the most significant enhancement observed in high-concentration treatments. This reflects an active physiological response to external stress. These findings are consistent with the results reported by Pignattelli et al. regarding the effects of polyethylene terephthalate (PET) stress on *Lepidium sativum* [79].

### 4.4. Effects of PS-NPs on MDA Content and Antioxidant Enzyme Activities

MDA is a key end-product of membrane lipid peroxidation, and its concentration sensitively reflects the degree of cellular oxidative damage, serving as a reliable indicator for evaluating oxidative stress levels in plants [76,80]. In this study, PS-NPs stress increased the MDA content in all three plant species, with the effect showing a positive correlation with nanoplastics concentration. This result is consistent with several studies, such as those on *Spirodela polyrhiza* [81] and *Salvinia natans* [82], which also exhibited rising MDA levels under PS-NPs stress. This phenomenon occurs because NPs stress triggers the intracellular accumulation of ROS, which attack the phospholipid bilayer of cell membranes and induce lipid peroxidation, thereby producing MDA [73]. The increase in MDA content further corroborates the observed decline in root activity and the rise in cell membrane permeability and Pro content.

Antioxidant enzymes constitute the core defense system for scavenging ROS and resisting oxidative damage [83], and their activity levels are frequently used to assess the degree of cellular damage in plants [84]. SOD converts highly toxic superoxide anion radicals(O_2_^-^) into H_2_O_2_ and O_2_, thereby mitigating cellular injury caused by ROS [85]. In this study, PS-NPs stress significantly increased SOD activity in all three plant species, with the magnitude of the increase being positively correlated with nanoplastics concentration. This finding is consistent with the conclusions of Jia et al. [18], who observed that 100 nm PS-NPs induced elevated SOD activity in *E. crassipes*, and also aligns with the results reported by Li et al. for *Vallisneria natans* [86]. These results suggest that NPs stress triggers the accumulation of superoxide radicals within plant tissues. From a temporal perspective, SOD activity in all three species exhibited an initial increase followed by a subsequent decrease, yet remained consistently higher than the control throughout the stress period. The initial rise in activity reflects a rapid and proactive response by the plants to nanoplastics-induced oxidative stress. The subsequent decline in activity may be attributed to a stabilized rate of ROS production, partial scavenging through alternative pathways, or weakened induction signals. Furthermore, it might be related to limited enzyme protein synthesis or oxidative damage to the enzymes themselves under chronic stress, reflecting a dynamic adaptive adjustment of the antioxidant defense system under sustained pressure [87,88]. Future long-term exposure experiments are warranted to further clarify the mechanisms responsible for the decline in SOD activity during the later stages of PS-NPs stress. It is necessary to determine whether this phenomenon represents an active adaptive response of the plant, an early indicator of irreversible damage to the antioxidant defense system, or an attenuation of ROS signaling induced by a reduction in effective PS-NPs concentrations.

POD and CAT play vital roles in the plant antioxidant system by decomposing H_2_O_2_ into harmless H_2_O and O_2_ [89]. In this study, the elevated activities of POD and CAT under PS-NPs stress indicate that, while activating SOD, the plants simultaneously enhanced their subsequent H_2_O_2_ scavenging capacity to resist nanoplastics-induced oxidative damage. These findings are consistent with the research by Pan et al. on *Spirodela polyrhiza* under PS-NPs stress [90]. In contrast, Yang et al. found that [91] low concentrations (0.5% and 1%) of PE treatment significantly enhanced the POD activity of *Avena sativa* L., while a high concentration (5%) of PE significantly reduced its POD activity. This discrepancy may be attributed to the excessive accumulation of H_2_O_2_ in different species or under specific stress conditions, which might have exceeded the regulatory threshold of POD and led to enzyme inhibition. Additionally, the dynamic changes in POD and CAT activities observed across the three species over time reflect the adaptive adjustment of antioxidant strategies under sustained stress.

Furthermore, the three plant species were treated with red fluorescently labeled nanoplastics in this study. The fluorescent dyes leached from the nanoplastics at a certain rate, and their potential biological toxicity should not be overlooked, as it may induce oxidative damage in plants [92].

### 4.5. Accumulation of PS-NPs in Plant Tissues

The site of lateral root emergence serves as the primary pathway for NPs to enter plants. The formation of lateral roots disrupts the Casparian strip defense of the endodermis, allowing NPs to cross the endodermis, enter the vascular bundle, and be transported upward to the stems and leaves via the transpiration stream [39,42,53]. The meristematic zone of the root tip represents another important pathway for NPs to enter plants. In this zone, the Casparian strip remains undifferentiated, and the cells undergo rapid growth and division. NPs adsorbed onto the cell surface can readily enter the intercellular spaces during cell division [16,63,93,94]. In addition, root wounds, leaf stomata and areas with a thin cuticle, also serve as pathways for NPs to enter plants [53,95,96,97,98].

In this study, laser confocal microscopy imaging revealed weak fluorescence signals in both the root maturation zone and the base of the petiole (Figure 9 and Figure S4), indicating that PS-NPs were taken up by the plants and translocated to the stem. The PS-NPs content in the roots of the three tested species was higher than in the leaves. This distribution is mainly related to the transport pathway of nanoplastics from roots to leaves within the plant [39,53,99]. Furthermore, at the same concentration level, the leaf accumulation of 20 nm PS-NPs was higher than that in the 100 nm treatment groups, while the opposite trend was observed in the roots. This may be because the larger 100 nm NPs are more easily trapped by the intercellular spaces of root tissues, leading to significant retention in the root system. Additionally, on day 3 of the culture period, *L. laevigatum* exhibited a strong capacity for absorption and accumulation under low-concentration treatments. However, as concentrations increased, *P. stratiotes* showed a higher accumulation capacity, potentially due to its specific root structure or physiological characteristics. Hou et al. [100] found that *P. stratiotes*, which possesses well-developed lateral roots, exhibited higher efficiency in the uptake and translocation of nanoparticles compared to *Alisma plantago-aquatica.* Olkhovych et al. [101] reported that *P. stratiotes* maintained a relatively stable photosynthetic system under nanoparticle stress and showed a higher potential for nanoparticles removal. In contrast, *E. crassipes* maintained lower overall PS-NPs concentrations across all treatments. This could be attributed to its rapid growth rate; since no additional NPs were supplemented during the culture period, the rapid increase in plant biomass likely diluted the internal NPs concentration, resulting in lower accumulation levels per unit of dry weight. Furthermore, the accumulation of PS-NPs in the three plants declined gradually with prolonged exposure time. This phenomenon may be attributed to the absence of new PS-NPs inputs during the experimental period, losses due to sedimentation, aggregation and adsorption onto container walls, along with reduced bioavailability [40,102,103].

Notably, although *Pistia stratiotes* exhibits a high potential for PS-NPs uptake and accumulation, it grows rapidly, reproduces vigorously, and is widely distributed in China, making it a highly invasive species. Therefore, when utilizing this plant for phytoremediation, it should be confined to closed water bodies or restricted using physical fixation techniques to prevent its uncontrolled spread.

## 5. Conclusions and Prospect

In this study, *L. laevigatum*, *P. stratiotes*, and *E. crassipes* were selected as test species to investigate the physiological and biochemical responses as well as the uptake and accumulation characteristics of three floating macrophytes exposed to PS-NPs at different concentrations (0.1, 1, and 10 mg·L^−1^) and particle sizes (20 and 100 nm). PS-NPs pollution exerted different effects on the physiological and biochemical characteristics of the three floating macrophyte species. With increasing concentrations and prolonged exposure, all three species exhibited decreases in chlorophyll content and root activity, alongside increases in cell membrane permeability. And the plants through accumulated osmotic regulatory substances and upregulated antioxidant enzyme activities to maintain osmotic balance and alleviate oxidative stress damage. In addition, the roots of all three floating macrophytes were capable of absorbing both sizes of PS-NPs and translocating them upward to the leaves. The distribution of PS-NPs in root tips, root maturation zones, and petiole bases displayed pronounced size and concentration dependence. The findings of this study contribute to a deeper understanding of the physiological and biochemical effects of nanoplastics pollution on aquatic plants, and provide reference data for the development of phytoremediation strategies for microplastic contaminated water bodies.

It should be noted that this study has several limitations. First, the use of monodisperse PS-NPs may oversimplify the interactions between nanoplastics and plants, as nanoplastics in natural environments are typically polydisperse. Second, a pulse exposure design was adopted, with PS-NPs added once at the beginning of the experiment and no further input over the following 45 days. Whereas NPs pollution in natural water bodies is usually continuous. Under continuous exposure, the physiological responses and uptake accumulation patterns of plants to NPs may differ. Therefore, future studies should consider the combined effects of NPs under continuous exposure conditions to more accurately assess plant responses to NPs stress and their uptake and accumulation capacity.

## Data Availability

The data that support the findings of this study are available on request from the corresponding author.

## Supplementary Materials

The following supporting information can be downloaded at: https://www.mdpi.com/article/10.3390/toxics14090802/s1, Text S1: Physiological indicators measurement methods; Text S2: Tracing of PS-NPs within plant tissues; Figure S1: Morphology and sizes of nanoplastics. (a) Morphology, (b) Sizes; Figure S2: Emission spectra of the PS-NPs. (a) Emission spectrum of red fluorescent PS-NPs under 535 nm excitation, (b) Emission spectrum of europium-doped PS-NPs under 365 nm excitation. S1: original spectral intensity; S1c: corrected spectral intensity; Figure S3: Standard curve for the concentration of europium-doped PS-NPs versus europium concentration. Left, 20 nm europium-doped PS-NPs standard curve; right, 100 nm europium-doped PS-NPs standard curve; Figure S4: Laser confocal microscopy image of three plant petioles: Table S1: Statistical power of the sample: Table S2: Results of two-way analyses of variance.

## References

[1] Thompson R.C., Olsen Y., Mitchell R.P., Davis A., Rowland S.J., John A.W.G., McGonigle D., Russell A.E. (2004). Lost at Sea: Where is all the plastic?. Science.

[2] (2016). EFSA Panel on Contaminants in the Food Chain (CONTAM). Presence of microplastics and nanoplastics in food, with particular focus on seafood. EFSA J..

[3] Allen S., Allen D., Phoenix V.R., Le Roux G., Durántez Jiménez P., Simonneau A., Binet S., Galop D. (2019). Atmospheric transport and deposition of microplastics in a remote mountain catchment. Nat. Geosci..

[4] Chen L., Zhou S., Zhang Q., Su B., Yin Q., Zou M. (2024). Global occurrence characteristics, drivers, and environmental risk assessment of microplastics in lakes: A meta-analysis. Environ. Pollut..

[5] Duan Q., Zhai B., Zhao C., Liu K., Yang X., Zhang H., Yan P., Huang L., Lee J., Wu W. (2024). Nationwide meta-analysis of microplastic distribution and risk assessment in China’s aquatic ecosystems, soils, and sediments. J. Hazard. Mater..

[6] Xiao J., Yang X., Zhang Z., Wang M., Yang Z., Zhang X. (2024). Research progress of the sources, distribution characteristics, and potential risks of microplastics in the global marine environment. Water Air Soil Pollut..

[7] Zhang K., Su J., Xiong X., Wu X., Wu C., Liu J. (2016). Microplastic pollution of lakeshore sediments from remote lakes in Tibet plateau, China. Environ. Pollut..

[8] Peeken I., Primpke S., Beyer B., Gütermann J., Katlein C., Krumpen T., Bergmann M., Hehemann L., Gerdts G. (2018). Arctic Sea ice is an important temporal sink and means of transport for microplastic. Nat. Commun..

[9] Zhang D., Liu X., Huang W., Li J., Wang C., Zhang D., Zhang C. (2020). Microplastic pollution in deep-sea sediments and organisms of the Western Pacific Ocean. Environ. Pollut..

[10] Choi J.S., Hong S.H., Park J. (2020). Evaluation of microplastic toxicity in accordance with different sizes and exposure times in the marine copepod *Tigriopus japonicus*. Mar. Environ. Res..

[11] Corinaldesi C., Canensi S., Dell’Anno A., Tangherlini M., Capua I.D., Varrella S., Willis T.J., Cerrano C., Danovaro R. (2021). Multiple impacts of microplastics can threaten marine habitat-forming species. Commun. Biol..

[12] Huang W., Song B., Liang J., Niu Q., Zeng G., Shen M., Deng J., Luo Y., Wen X., Zhang Y. (2021). Microplastics and associated contaminants in the aquatic environment: A review on their ecotoxicological effects, trophic transfer, and potential impacts to human health. J. Hazard. Mater..

[13] Eze C.G., Nwankwo C.E., Dey S. (2024). Food chain microplastics contamination and impact on human health: A review. Environ. Chem. Lett..

[14] Song U., Kim J., Rim H. (2023). Assessing phytotoxicity of microplastics on aquatic plants using fluorescent microplastics. Environ. Sci. Pollut. Res..

[15] Yuan W., Xu E.G., Li L., Zhou A., Peijnenburg W.J.G.M., Grossart H., Liu W., Yang Y. (2023). Tracing and trapping micro- and nanoplastics: Untapped mitigation potential of aquatic plants?. Water Res..

[16] Zhao Y., Hu C., Wang X., Cheng H., Xing J., Li Y., Wang L., Ge T., Du A., Wang Z. (2024). Water spinach (*Ipomoea aquatica* F.) effectively absorbs and accumulates microplastics at the micron level—A study of the co-exposure to microplastics with varying particle sizes. Agriculture.

[17] Zhang J., Huang D., Deng H., Zhang J. (2022). Responses of submerged plant *Vallisneria* natans growth and leaf biofilms to water contaminated with microplastics. Sci. Total Environ..

[18] Jia H., Yu H., Li J., Qi J., Zhu Z., Hu C. (2023). Trade-off of abiotic stress response in floating macrophytes as affected by nanoplastic enrichment. J. Hazard. Mater..

[19] Chen X., Ma H., Kong C., Pan T., Gao D., Liao H., Wang J. (2024). Bioaccumulation of polystyrene nanoplastics and BDE-209 induced oxidative stress, photosynthesis and growth impairments in floating fern *Salvinia natans*. Sci. Total Environ..

[20] Yao X., Lin Z., Chen W., Pan Z., Hou L., Li J., Li D., Liu W., Hu K. (2024). Toxic effects of microplastics (MPs) on aquatic plants and phytoremediation potential in freshwater environments. J. Water Process Eng..

[21] Liu Y., Wei L., Yu H., Cao X., Peng J., Liu H., Qu J. (2022). Negative impacts of nanoplastics on the purification function of submerged plants in constructed wetlands: Responses of oxidative stress and metabolic processes. Water Res..

[22] Wang Y., Zhu X., Lao Y., Lv X., Tao Y., Huang B., Wang J., Zhou J., Cai Z. (2016). TiO_2_ nanoparticles in the marine environment: Physical effects responsible for the toxicity on algae *Phaeodactylum tricornutum*. Sci. Total Environ..

[23] Yang G., Zheng M., Liao H., Tan A., Feng D., Lv S. (2022). Influence of cadmium and microplastics on physiological responses, ultrastructure and rhizosphere microbial community of duckweed. Ecotoxicol. Environ. Saf..

[24] Ceschin S., Mariani F., Di Lernia D., Venditti I., Pelella E., Iannelli M.A. (2023). Effects of microplastic contamination on the aquatic plant *Lemna minuta* (least duckweed). Plants.

[25] Parsai T., Figueiredo N., Dalvi V., Martins M., Malik A., Kumar A. (2022). Implication of microplastic toxicity on functioning of microalgae in aquatic system. Environ. Pollut..

[26] Zhang S., Han L., Peng J., Liu R., Liu H., Qu J. (2024). The mechanism of a submerged aquatic plant to various size of micro-nano plastics stress in ecological constructed wetland. Chem. Eng. J..

[27] Zhang X., Li W., Xue W., Adomako M.O., Tang M., He L., Yu F. (2024). Effects of soil microplastic heterogeneity on plant growth vary with species and microplastic types. Sci. Total Environ..

[28] Zhang X., Lu L., Jin Y., Yang J., Feng C., Xu K., Wei D. (2025). Effect of different biodegradability of microplastics on an emergent aquatic plant in constructed wetlands: Responses of tolerance and metabolic function. J. Environ. Chem. Eng..

[29] Mao H., Yang H., Xu Z., Yang Y., Zhang X., Huang F., Wei L., Li Z. (2023). Microplastics and co-pollutant with ciprofloxacin affect interactions between free-floating macrophytes. Environ. Pollut..

[30] Niklas K.J., Cobb E.D. (2017). The evolutionary ecology (evo-eco) of plant asexual reproduction. Evol. Ecol..

[31] Žižková E., Kubeš M., Dobrev P.I., Přibyl P., Šimura J., Zahajská L., Záveská Drábková L., Novák O., Motyka V. (2017). Control of cytokinin and auxin homeostasis in cyanobacteria and algae. Ann. Bot..

[32] Wang Z., Sun C., Li F., Chen L. (2021). Fatigue resistance, re-usable and biodegradable sponge materials from plant protein with rapid water adsorption capacity for microplastics removal. Chem. Eng. J..

[33] Yogarathinam L.T., Usman J., Othman M.H.D., Ismail A.F., Goh P.S., Gangasalam A., Adam M.R. (2022). Low-cost silica based ceramic supported thin film composite hollow fiber membrane from guinea corn husk ash for efficient removal of microplastic from aqueous solution. J. Hazard. Mater..

[34] Peydayesh M., Suta T., Usuelli M., Handschin S., Canelli G., Bagnani M., Mezzenga R. (2021). Sustainable removal of microplastics and natural organic matter from water by coagulation-flocculation with protein amyloid fibrils. Environ. Sci. Technol..

[35] Gao R., Liu R., Sun C. (2022). A marine fungus *Alternaria alternata* FB1 efficiently degrades polyethylene. J. Hazard. Mater..

[36] Zhao Y., Yu C., Chen P., Mou P., Chen J., Gao G., Wang X., Zhu A., Chen K. (2024). Study on remediation of cadmium contaminated paddy field by ramie (*Boehmeria nivea* L.) floating island and its supporting technology. Environ. Res..

[37] Zhou T., An Q., Zhang L., Wen C., Yan C. (2024). Phytoremediation for antibiotics removal from aqueous solutions: A meta-analysis. Environ. Res..

[38] Sundbæk K.B., Koch I.D.W., Villaro C.G., Rasmussen N.S., Holdt S.L., Hartmann N.B. (2018). Sorption of fluorescent polystyrene microplastic particles to edible seaweed Fucus vesiculosus. J. Appl. Phycol..

[39] Li L., Luo Y., Li R., Zhou Q., Peijnenburg W.J.G.M., Yin N., Yang J., Tu C., Zhang Y. (2020). Effective uptake of submicrometre plastics by crop plants via a crack-entry mode. Nat. Sustain..

[40] Cheng Y., Wang H. (2022). Highly effective removal of microplastics by microalgae *Scenedesmus abundans*. Chem. Eng. J..

[41] Kim Y., Park K., Bak J., Choi S. (2024). *Iris pseudacorus* and *Lythrum anceps* as plants supporting the process of removing microplastics from aquatic environments-preliminary research. Horticulturae.

[42] Yin J., Zhu T., Li X., Wang F., Xu G. (2025). Phytoremediation of microplastics by water hyacinth. Environ. Sci. Ecotechnol..

[43] Cancian L., Camargo A., Silva G. (2009). Growth of *Pistia stratiotes* in different temperature and photoperiod regimes. Acta Bot. Bras..

[44] Arán D.S., Harguinteguy C.A., Fernandez-Cirelli A., Pignata M.L. (2017). Phytoextraction of Pb, Cr, Ni, and Zn using the aquatic plant *Limnobium laevigatum* and its potential use in the treatment of wastewater. Environ. Sci. Pollut. Res..

[45] Acosta Rodríguez I., Rodríguez Pérez A., Pacheco Castillo N.C., Enríquez Domínguez E., Cárdenas González J.F., Martínez Juárez V.M. (2021). Removal of cobalt (II) from waters contaminated by the biomass of *Eichhornia crassipes*. Water.

[46] Eid E.M., Shaltout K.H., Almuqrin A.H., Aloraini D.A., Khedher K.M., Taher M.A., Alfarhan A.H., Picó Y., Barcelo D. (2021). Uptake prediction of nine heavy metals by *Eichhornia crassipes* grown in irrigation canals: A biomonitoring approach. Sci. Total Environ..

[47] De Araujo L.G., Vieira L.C., Canevesi R.L.S., Da Silva E.A., Watanabe T., De Padua Ferreira R.V., Marumo J.T. (2022). Biosorption of uranium from aqueous solutions by *Azolla sp.* and *Limnobium laevigatum*. Environ. Sci. Pollut. Res..

[48] Ahmed M.T., Alrumman S.A., Singh A.K., Kumar P., Singh J. (2025). Kinetic assessment of plant growth and pollutant removal by water lettuce (*Pistia stratiotes* L.) from integrated industrial wastewater: A lab-scale study. Water Air Soil Pollut..

[49] Choong W.S., Hadibarata T., Yuniarto A., Tang K.H.D., Abdullah F., Syafrudin M., Al Farraj D.A., Al-Mohaimeed A.M. (2021). Characterization of microplastics in the water and sediment of Baram River estuary, Borneo Island. Mar. Pollut. Bull..

[50] Liong R.M.Y., Hadibarata T., Yuniarto A., Tang K.H.D., Khamidun M.H. (2021). Microplastic occurrence in the water and sediment of Miri River Estuary, Borneo Island. Water Air Soil Pollut..

[51] Ermolin M.S., Ivaneev A.I., Savonina E.Y., Dzhenloda R.K. (2025). Extraction of microplastics from river water in a rotating coiled column using a water–oil system. J. Anal. Chem..

[52] Mendrik F., Hackney C.R., Cumming V.M., Waller C., Hak D., Dorrell R., Hung N.N., Parsons D.R. (2025). The transport and vertical distribution of microplastics in the Mekong River, SE Asia. J. Hazard. Mater..

[53] Yang C., Yin L., Guo Y., Han T., Wang Y., Liu G., Maqbool F., Xu L., Zhao J. (2023). Insight into the absorption and migration of polystyrene nanoplastics in *Eichhornia crassipes* and related photosynthetic responses. Sci. Total Environ..

[54] Zheng X., Wu H., Li G., Li Q., Zheng Z., Zhang W. (2025). Unraveling the toxic mechanisms of microplastics in aquatic ecosystem: A case study on *Vallisneria natans* and *Myriophyllum verticillatum*. Environ. Pollut..

[55] Wang X. (2006). Principles and Techniques of Plant Physiological and Biochemical Experiments.

[56] Tang S., Luo C. (2012). Experimental Course of Plant Physiology.

[57] Mueller Y., Wernicke T., Pittroff M., Witzig C., Storck F., Klinger J., Zumbuelte N. (2020). Microplastic analysis-are we measuring the same? Results on the first global comparative study for microplastic analysis in a water sample. Anal. Bioanal. Chem..

[58] Zhang C., Jian M., Chen Y., Chen Q., He X., Cong M., Yang W. (2021). Effects of polystyrene microplastics (PS-MPs) on the growth, physiology, and biochemical characteristics of *Hydrilla verticillate*. Chin. J. Appl. Ecol..

[59] Lu C., Jiang C., Feng Y., Lei J., Lei S. (2024). Growth and physiological response of two submerged plants to polylactic acid microplastic stresses. Acta Sci. Circumst..

[60] Wang Y., Xiang L., Wang F., Wang Z., Bian Y., Gu C., Wen X., Kengara F.O., Schäffer A., Jiang X. (2022). Positively charged microplastics induce strong lettuce stress responses from physiological, transcriptomic, and metabolomic perspectives. Environ. Sci. Technol..

[61] Wang J., Zhu J., Zheng Q., Wang D., Wang H., He Y., Wang J., Zhan X. (2023). In vitro wheat protoplast cytotoxicity of polystyrene nanoplastics. Sci. Total Environ..

[62] Öztürk B.Y. (2025). Biodegradable PBAT-derived microplastics induce metabolic and ultrastructural alterations in *Gloeocystis ampla* (Kützing) Rabenhorst. Algal Res..

[63] Zhang C., Yue N., Li X., Shao H., Wang J., An L., Jin F. (2023). Potential translocation process and effects of polystyrene microplastics on strawberry seedlings. J. Hazard. Mater..

[64] Kalčíkovái G., Žgajnar Gotvajn A., Kokalj A.J., Kladnik A. (2017). Impact of polyethylene microbeads on the floating freshwater plant duckweed *Lemna minor*. Environ. Pollut..

[65] Wang Y., Tan L., Meng F., Xu H., Wang J. (2026). Multiple indicator assessment of the toxicity of micro-and nanoplastics on the harmful algae *Heterosigma akashiwo*. Process Saf. Environ. Prot..

[66] Yu H., Peng J., Cao X., Wang Y., Zhang Z., Xu Y., Qi W. (2021). Effects of microplastics and glyphosate on growth rate, morphological plasticity, photosynthesis, and oxidative stress in the aquatic species *Salvinia cucullata*. Environ. Pollut..

[67] Taylor S.E., Pearce C.I., Sanguinet K.A., Hu D., Chrisler W.B., Kim Y., Wang Z., Flury M. (2020). Polystyrene nano- and microplastic accumulation at Arabidopsis and wheat root cap cells, but no evidence for uptake into roots. Environ. Sci. Nano.

[68] Parkinson S.J., Tungsirisurp S., Joshi C., Richmond B.L., Gifford M.L., Sikder A., Lynch I., O’Reilly R.K., Napier R.M. (2022). Polymer nanoparticles pass the plant interface. Nat. Commun..

[69] Xiao F., Feng L., Sun X., Wang Y., Wang Z., Zhu F., Yuan X. (2022). Do polystyrene nanoplastics have similar effects on duckweed (*Lemna minor* L.) at environmentally relevant and observed-effect concentrations?. Environ. Sci. Technol..

[70] Yao M., Mu L., Gao Z., Hu X. (2023). Persistence of algal toxicity induced by polystyrene nanoplastics at environmentally relevant concentrations. Sci. Total Environ..

[71] Song J., Yang K., Ding A., Jin N., Sun Y., Zhang D. (2025). Antagonistic effects of polystyrene microplastics and tetracycline on *Chlorella pyrenoidosa* as revealed by infrared spectroscopy coupled with multivariate analysis. J. Hazard. Mater..

[72] Wang J., Tan L., Li Q., Wang J. (2024). Toxic effects of nSiO_2_ and mPS on diatoms *Nitzschia closterium f.* minutissima. Mar. Environ. Res..

[73] Wong-Ekkabut J., Xu Z., Triampo W., Tang I.M., Tieleman D.P., Monticelli L. (2007). Effect of lipid peroxidation on the properties of lipid bilayers: A molecular dynamics study. Biophys. J..

[74] Couée I., Sulmon C., Gouesbet G., Amrani A.E. (2006). Involvement of soluble sugars in reactive oxygen species balance and responses to oxidative stress in plants. J. Exp. Bot..

[75] Zhou C., Lu C., Mai L., Bao L., Liu L., Zeng E. (2021). Response of rice (*Oryza sativa* L.) roots to nanoplastic treatment at seedling stage. J. Hazard. Mater..

[76] Li S., Wang P., Zhang C., Zhou X., Yin Z., Hu T., Hu D., Liu C., Zhu L. (2020). Influence of polystyrene microplastics on the growth, photosynthetic efficiency and aggregation of freshwater microalgae *Chlamydomonas reinhardtii*. Sci. Total Environ..

[77] Nugraha A., Idris F., Apriadi T., Dhevanda C., Azis M.Y., Hafsar K. (2025). Impact of microplastic exposure on the health of tropical seagrass (*Enhalus acoroides*) seedlings. Mar. Pollut. Bull..

[78] Mudgal V., Madaan V., Mudgal A. (2010). Biochemical mechanisms of salt tolerance in plants: A review. Int. J. Bot..

[79] Pignattelli S., Broccoli A., Piccardo M., Terlizzi A., Renzi M. (2021). Effects of polyethylene terephthalate (PET) microplastics and acid rain on physiology and growth of *Lepidium sativum*. Environ. Pollut..

[80] Ayala A., Munoz M.F., Arguelles S. (2014). Lipid peroxidation: Production, metabolism, and signaling mechanisms of malondialdehyde and 4-hydroxy-2-nonenal. Oxidative Med. Cell. Longev..

[81] Zhou A., Zhao Y., Liu M., Suyamud B., Yuan W., Yang Y. (2023). Occurrence and risk assessment of microplastics in the Lhasa River-a remote plateau river on the Qinghai-Tibet Plateau, China. Environ. Monit. Assess..

[82] Chen G., Pan T., Gao D., Liao H., Wang J. (2024). Enhanced competitiveness of *Spirodela polyrhiza* in co-culture with *Salvinia natans* under combined exposure to polystyrene nanoplastics and polychlorinated biphenyls. Sci. Total Environ..

[83] Abele D., Vazquez-Medina J.P., Zenteno-Savín T. (2011). Introduction to oxidative stress in aquatic ecosystems. Oxidative Stress in Aquatic Ecosystems.

[84] Mittler R. (2002). Oxidative stress, antioxidants and stress tolerance. Trends Plant Sci..

[85] Gill S.S., Anjum N.A., Gill R., Yadav S., Hasanuzzaman M., Fujita M., Mishra P., Sabat S.C., Tuteja N. (2015). Superoxide dismutase—Mentor of abiotic stress tolerance in crop plants. Environ. Sci. Pollut. Res..

[86] Li Q., Xiao Y., Zhang W., Li S., Liu J., Yu Y., Wen Y., Zhang Y., Lei N., Wang Q. (2023). Single and combined toxicity effects of microplastics and perfluorooctanoic acid on submerged macrophytes and biofilms. Sci. Total Environ..

[87] Lian J., Wu J., Zeb A., Zheng S., Ma T., Peng F., Tang J., Liu W. (2020). Do polystyrene nanoplastics affect the toxicity of cadmium to wheat (*Triticum aestivum* L.)?. Environ. Pollut..

[88] Gao D., Liao H., Junaid M., Chen X., Kong C., Wang Q., Pan T., Chen G., Wang X., Wang J. (2023). Polystyrene nanoplastics’ accumulation in roots induces adverse physiological and molecular effects in water spinach *Ipomoea aquatica* Forsk. Sci. Total Environ..

[89] Asada K. (1999). The water-water cycle in chloroplasts: Scavenging of active oxygens and dissipation of excess photons. Annu. Rev. Plant Biol..

[90] Pan T., Chen X., Kong C., Gao D., Liu W., Liao H., Junaid M., Wang J. (2023). Single and combined toxicity of polystyrene nanoplastics and PCB-52 to the aquatic duckweed *Spirodela polyrhiza*. Sci. Total Environ..

[91] Yang Z., Zhao L., Tan S., Mao P., Wang Q., Ma W. (2025). Effects of polyethylene and polystyrene microplastics on oat (*Avena sativa* L.) growth and physiological characteristics. Plants.

[92] Malafaia G., Da Luz T.M., Ahmed M.A.I., Karthi S., Da Costa Araújo A.P. (2022). When toxicity of plastic particles comes from their fluorescent dye: A preliminary study involving neotropical Physalaemus cuvieri tadpoles and polyethylene microplastics. J. Hazard. Mater. Adv..

[93] Liu Y., Guo R., Zhang S., Sun Y., Wang F. (2022). Uptake and translocation of nano/microplastics by rice seedlings: Evidence from a hydroponic experiment. J. Hazard. Mater..

[94] Liu C., Jiao Y., Guo J., Li B., Gu C., Qian T., Liu X. (2024). Tracing the entry process of submicrometre plastics in soybean sprouts by leaf-derived fluorescent carbon dots. J. Hazard. Mater..

[95] Lian J., Liu W., Meng L., Wu J., Chao L., Zeb A., Sun Y. (2021). Foliar-applied polystyrene nanoplastics (PSNPs) reduce the growth and nutritional quality of lettuce (*Lactuca sativa* L.). Environ. Pollut..

[96] Sun H., Lei C., Xu J., Li R. (2021). Foliar uptake and leaf-to-root translocation of nanoplastics with different coating charge in maize plants. J. Hazard. Mater..

[97] Li Y., Zhang J., Xu L., Li R., Zhang R., Li M., Ran C., Rao Z., Wei X., Chen M. (2025). Leaf absorption contributes to accumulation of microplastics in plants. Nature.

[98] Yin J., Li X., Cui F., Yu Y., Xing B., Wang F., Xu G. (2026). Root wounds facilitate the uptake of microplastics in crop plants. Nat. Commun..

[99] Sun X., Yuan X., Jia Y., Feng L., Zhu F., Dong S., Liu J., Kong X., Tian H., Duan J. (2020). Differentially charged nanoplastics demonstrate distinct accumulation in *Arabidopsis thaliana*. Nat. Nanotechnol..

[100] Hou L., Liang Q., Yang G., Gao L., Liu X. (2023). Translocation of TiO_2_ nanoparticles enhances phosphorus uptake by wetland plants: Evidence from Pistia stratiotes and Alisma plantago-aquatica. J. Environ. Manag..

[101] Olkhovych O., Svietlova N., Konotop Y., Karaushu O., Hrechishkina S. (2016). Removal of metal nanoparticles colloidal solutions by water plants. Nanoscale Res. Lett..

[102] Besseling E., Quik J.T., Sun M., Koelmans A.A. (2017). Fate of nano- and microplastic in freshwater systems: A modeling study. Environ. Pollut..

[103] Li S., Liu H., Gao R., Abdurahman A., Dai J., Zeng F. (2018). Aggregation kinetics of microplastics in aquatic environment: Complex roles of electrolytes, pH, and natural organic matter. Environ. Pollut..

