# Supplementary material for: Physiological Responses and Absorption Removal Effects of Three Floating Macrophytes to Polystyrene Nanoplastics Pollution
Source: Toxics. 2026 Sep 10;14(9):802. doi: 10.3390/toxics14090802 (PMC13611539; doi:10.3390/toxics14090802)
Supplement: Supplementary file 1 [file toxics-14-00802-s001.zip › toxics-4545236-supplementary.pdf]

Supplementary Material

Text S1: Physiological indicators measurement methods

Chlorophyll Content

Chlorophyll was extracted from 0.1 g of fresh leaf tissue (excluding the midrib). The samples were homogenized and incubated in 5 mL of pre-cooled 95% ethanol in the dark at room temperature for 24–36 h until the tissue became completely white. The extract was then centrifuged at 4,000 r·min^{-1} for 10 min (Centrifuge 5804R, Eppendorf, Hamburg, Germany). Absorbance of the supernatant was measured at 665 nm and 649 nm using 95% ethanol as a blank (UV-1800PC spectrophotometer, Mapada Instruments, Shanghai, China). The concentrations (mg·L^{-1}) of chlorophyll a (*Chl a*), chlorophyll b (*Chl b*), and total chlorophyll (*Chl*) were calculated using the following equations [1]:

$$Chl\ a = 13.95 \times A_{665} - 6.88 \times A_{649} \quad (S1)$$

$$Chl\ b = 24.96 \times A_{649} - 7.32 \times A_{665} \quad (S2)$$

$$Chl\ = 18.08 \times A_{649} - 6.63 \times A_{665} \quad (S3)$$

$$\text{Chlorophyll content (mg} \cdot \text{g}^{-1}\ \text{FW)} = \frac{\text{chlorophyll concentration} \times \text{extract volume} \times \text{dilution factor}}{\text{sample fresh weight}} \quad (S4)$$

Root Activity

Root activity was determined using the triphenyl tetrazolium chloride (TTC) method [1]. Briefly, 0.2 g of fresh root tips were immersed in a mixture of 5 mL of 0.4% TTC solution and 5 mL of phosphate buffer (pH 7.0), then incubated in the dark at 37 °C for 2 h. The reaction was terminated by adding 2 mL of H_2SO_4. The roots were then extracted with 10 mL of ethyl acetate and homogenized to measure the absorbance at 485 nm. A blank control was prepared by adding H_2SO_4 before incubation. The amount of reduced tetrazolium was determined using a standard curve. Root activity, expressed as the reduction intensity of TTC per unit fresh weight per hour (mg·g^{-1}·h^{-1}), was calculated as follows:

$$\text{Root activity} = \frac{\text{C}}{\text{W} \times \text{t}} \quad (S5)$$

Where *C* is the mass of reduced tetrazolium (mg), *W* is the sample fresh weight (g) and *t* is the incubation time (h).

Cell Membrane Permeability

Cell membrane permeability was evaluated by measuring relative electric conductivity. Fresh leaves were rinsed with deionized water and blotted dry. Leaf discs were collected using a 0.5 cm diameter punch (avoiding the midrib), and 0.2 g of the mixed tissue was placed into a test tube containing 20 mL of deionized water. The samples were shaken in a constant temperature shaker at 25 °C for 1 h (ZQTY-70, Shanghai Zhichu Instruments Co., Ltd., Shanghai, China), after which the initial conductivity (*C1*) was measured using a conductivity meter (Orion Star A322, Thermo Fisher Scientific, USA). Subsequently, the tubes were placed in a boiling water bath for 10 min to release all electrolytes. After cooling to room temperature, the final conductivity (*C2*) was recorded. The conductivity of the deionized water (*C0*) was also measured as a background. Cell membrane permeability was calculated using the following equation[1]:

$$\text{Cell membrane permeability} = \frac{C1 - C0}{C2 - C0} \times 100\% \quad (S6)$$

Soluble Sugar Concentration and Malondialdehyde Content

Soluble sugar (SS) concentrations and malondialdehyde (MDA) contents were determined using the thiobarbituric acid (TBA) method[2]. Briefly, 0.3 g of fresh leaf tissue was homogenized in 10 mL of 10% trichloroacetic acid (TCA). The homogenate was centrifuged at 4,000 r·min^{-1} for 10 min. A 2 mL aliquot of the

supernatant (with 2 mL of distilled water as a blank) was mixed with 2 mL of 0.6% TBA solution. The mixture was sealed and heated in a boiling water bath for 15 min, then rapidly cooled in an ice-water bath. After a final centrifugation at 4,000 r·min^{-1} for 10 min, the absorbance of the supernatant was measured at 450 nm, 532 nm and 600 nm (UV-1800PC spectrophotometer, Mapada Instruments, Shanghai, China).

SS and MDA concentrations were calculated as follows:

$$\mathrm{SS}\ (\mathrm{mmol}\cdot\mathrm{L}^{-1}) = 11.71 \times A_{450} \tag{S7}$$

$$\mathrm{MDA}\ (\mu\mathrm{mol}\cdot\mathrm{L}^{-1}) = 6.45(A_{532} - A_{600}) - 0.56A_{450} \tag{S8}$$

The final MDA content (nmol·g^{-1} FW) was calculated using the equation:

$$\text{MDA content} = \frac{MDA\ concentration \times ts \times V}{W} \tag{S9}$$

Where *ts* is the dilution factor, *V* is the total volume of the extract (10 mL), and *W* is the sample fresh weight (g).

Soluble Protein Content

Soluble protein (SP) content was determined using the Coomassie Brilliant Blue G-250 staining method. Briefly, 0.5 g of fresh leaf tissue was homogenized in 8 mL of distilled water. The mixture was allowed to stand at room temperature for 1 h to ensure complete extraction, followed by centrifugation at 4,000 r·min^{-1} for 20 min (Centrifuge 5804R, Eppendorf, Hamburg, Germany). The supernatant was transferred to a 10 mL volumetric flask and diluted to the mark with distilled water to obtain the sample extract. A 0.1 mL aliquot of the extract was mixed with 0.9 mL of 0.15 mol·L^{-1} NaCl and 5 mL of Coomassie Brilliant Blue solution. After shaking and standing for 3 min, the absorbance was measured at 595 nm against a blank (UV-1800PC spectrophotometer, Mapada Instruments, Shanghai, China). The protein mass was determined using a standard curve. The SP content (mg·g^{-1} FW) was calculated as follows[2]:

$$\text{SP content} = \frac{m \times V_T \times F}{m_0 \times V_s \times 1000} \tag{S10}$$

Where *m* is the protein mass obtained from the standard curve (μg), V_T is the total volume of the extract (mL), *F* is the dilution factor, m_0 is the sample fresh weight (g), and V_S is the volume of the aliquot used for the assay (mL).

Antioxidant Enzyme Activities and Proline Content

O_2^- can reduce nitroblue tetrazolium to form blue formazan, which has an absorption peak at 560 nm. Superoxide dismutase (SOD) can scavenge O_2^- and inhibit the formation of formazan, allowing SOD activity to be calculated by measuring the change in absorbance. Peroxidase (POD) catalyzes the oxidation of specific substrates by H_2O_2, resulting in a characteristic optical absorption at 470 nm. POD activity is calculated by measuring the increase in absorbance per unit time at 470 nm. Catalase (CAT) decomposes H_2O_2, causing the absorbance of the reaction system at 240 nm to decrease over time. CAT activity is reflected by measuring the rate of decrease in absorbance at 240 nm. The proline (Pro) extract was reacted with an acidic ninhydrin solution to produce a red chromophore. Following extraction with toluene, the absorbance of the organic phase was measured at 520 nm to determine the proline content. Fresh leaf tissue (0.1 g) was homogenized in 1 mL of extraction buffer. The mixture was centrifuged at 8,000 r·min^{-1} for 10 min at 4 °C (Centrifuge 5804R, Eppendorf, Hamburg, Germany), and the resulting supernatant was collected for subsequent assays. The activities of SOD (EC 1.15.1.1), POD (EC 1.11.1.7), and CAT (EC 1.11.1.6) were determined using commercial assay kits (Solarbio, Beijing, China). Pro content was measured using an assay kit (Komson, Nanjing, Jiangsu province, China). All procedures were performed strictly according to the manufacturers' protocols.

Text. S2: Tracing of PS-NPs within Plant Tissues.

After 45 days, plants were harvested and washed with deionized water. The root maturation zone and petiole base were cut into small pieces, fixed in 70% FAA for 24 h, dehydrated in 10-30% sucrose solutions, and embedded with OTC. Cross-sections (40 μm) of roots and petioles were cut using a cryostat (EM UC7, Leica Microsystems, Wetzlar, Germany). Sections were placed on slides and imaged for red fluorescent PS-NPs particles by laser confocal scanning microscopy (LSM710, Zeiss, Germany) with excitation at 535 nm, emission at 610 nm, and an exposure time of 100 ms [3].

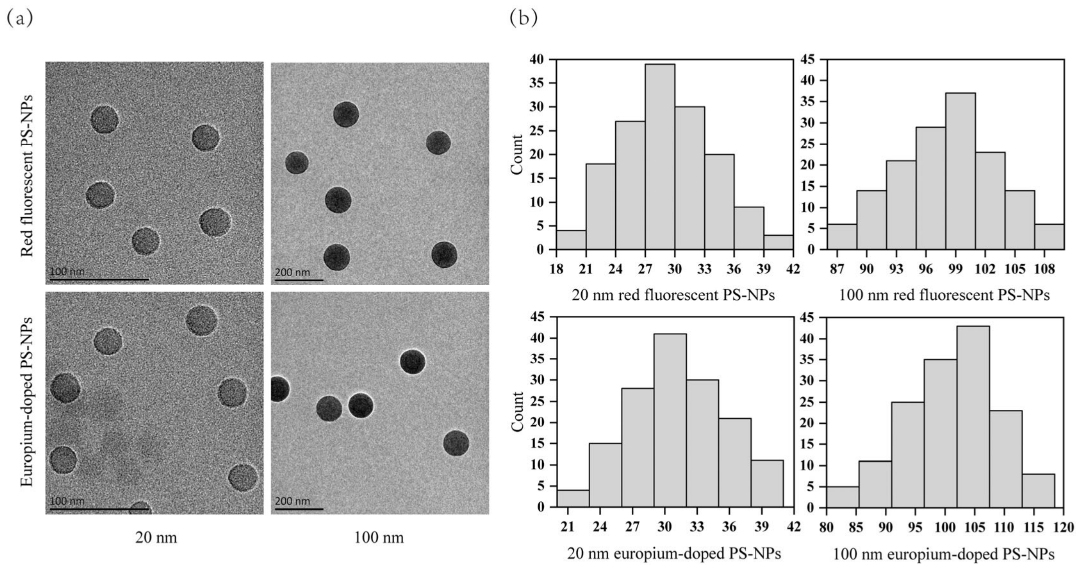

Figure S1. Morphology and sizes of nanoplastics. (a) Morphology, (b) Sizes.

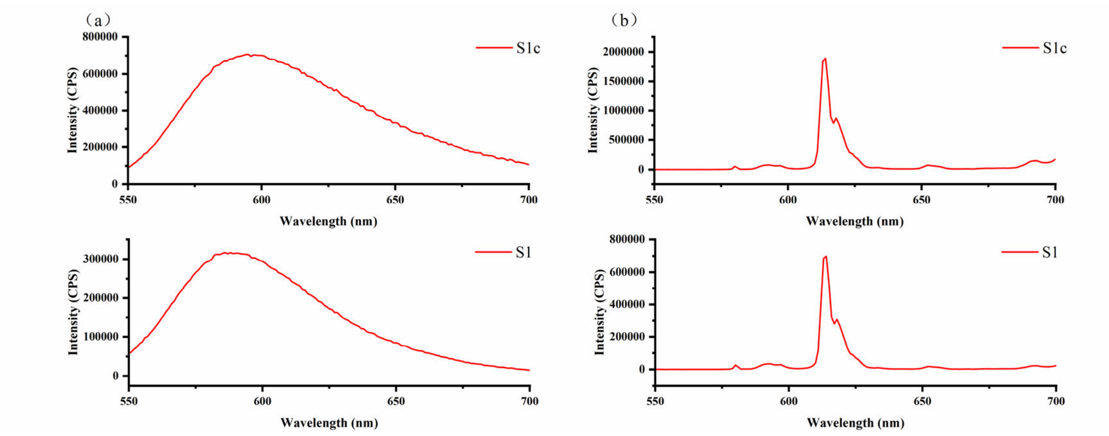

Figure S2. Emission spectra of the PS-NPs. (a) Emission spectrum of red fluorescent PS-NPs under 535 nm excitation, (b) Emission spectrum of europium-doped PS-NPs under 365 nm excitation. S1: original spectral intensity; S1c: corrected spectral intensity.

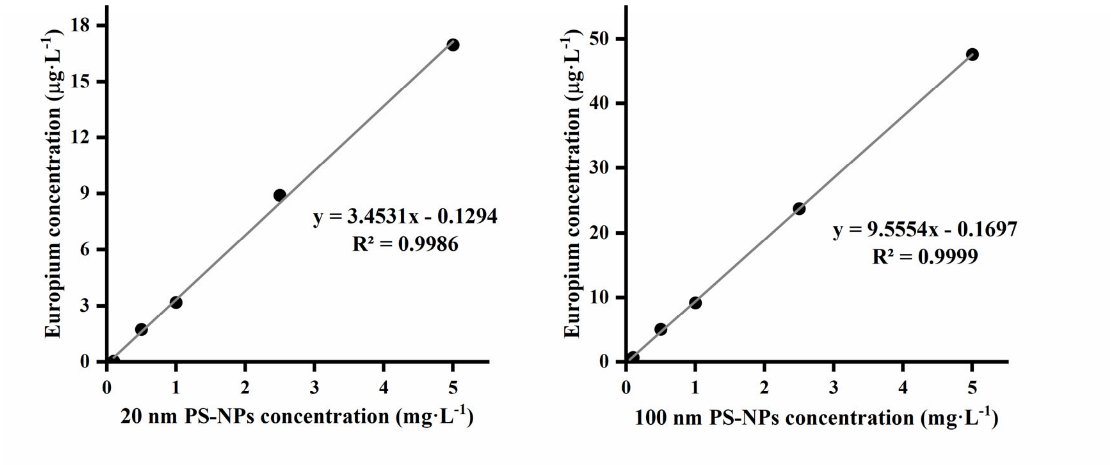

Figure S3. Standard curve for the concentration of europium-doped PS-NPs versus europium concentration. Left, 20 nm europium-doped PS-NPs standard curve; right, 100 nm europium-doped PS-NPs standard curve.

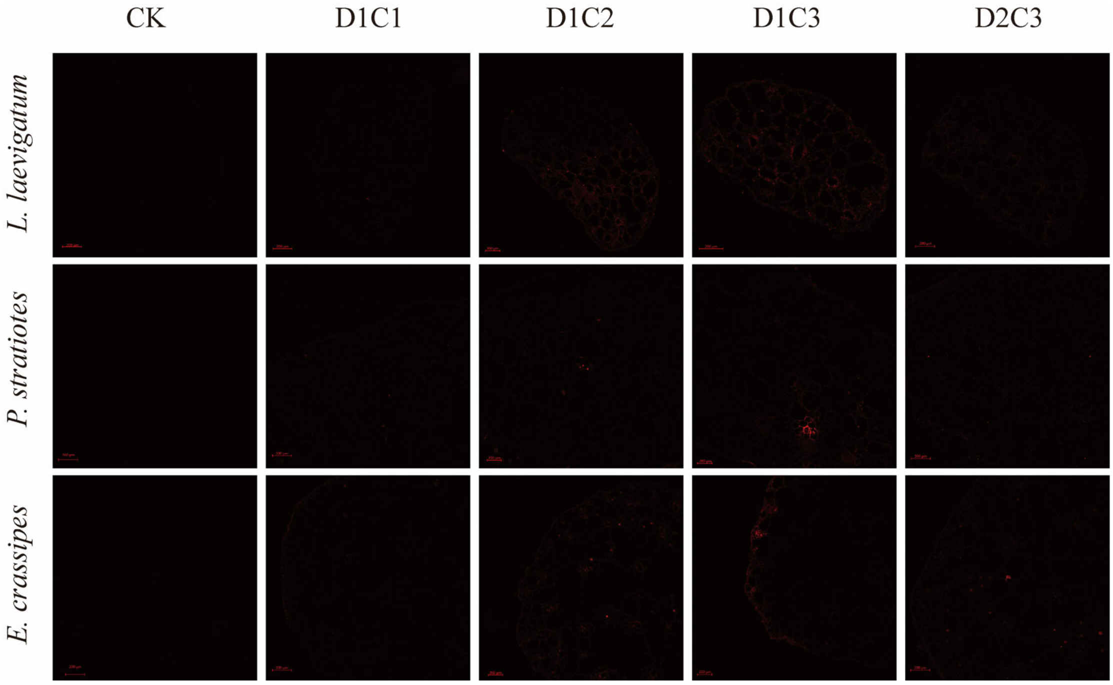

Figure S4. Laser confocal microscopy image of three plant petioles.

Table S1. Statistical power of the sample.

		Chl a			Chl b			Chl			Cell membrane permeability			SS		
		F	Effect Size	Power	F	Effect Size	Power	F	Effect Size	Power	F	Effect Size	Power	F	Effect Size	Power
	Treatment	24.545	1.279	1	24.545	2.558	1	185.402	7.031	1	13.337	1.886	0.998	4.903	1.143	0.813
L. laevigatum	Time	117.008	2.419	1	117.008	5.586	1	1.679	0.669	0.345	41.847	3.341	1	137.501	6.055	1
	Treatment×Time	1.888	0.614	0.674	1.888	0.71	0.385	23.287	2.492	1	2.588	0.831	0.513	2.525	0.821	0.502
	Treatment	27.612	1.357	1	27.612	2.714	1	81.524	4.663	1	14.328	1.955	0.999	26.239	2.645	1
P. stratiotes	Time	72.072	2.192	1	72.072	4.384	1	2.512	0.818	0.5	290.164	8.796	1	309.549	9.086	1
	Treatment×Time	3.516	0.419	0.753	3.516	0.968	0.658	13.816	1.919	0.999	3.219	0.926	0.615	7.339	1.399	0.945
	Treatment	12.221	1.563	1	12.221	1.805	0.997	272.161	8.519	1	17.746	2.175	1	5.321	1.191	0.846
E. crassipes	Time	225.252	3.356	1	225.252	7.75	1	4.607	1.108	0.786	160.484	6.542	1	3591.013	30.945	1
	Treatment×Time	3.906	0.884	0.971	3.906	1.021	0.709	91.142	4.93	1	5.687	1.231	0.871	14.926	1.995	0.999

Table S1. (Continued)

		SP			Root activity			Pro			MDA			SOD		
		F	Effect Size	Power	F	Effect Size	Power	F	Effect Size	Power	F	Effect Size	Power	F	Effect Size	Power
	Treatment	4.555	1.102	0.781	91.142	4.93	1	180.534	6.938	1	76.104	4.505	1	507.373	11.632	1
L. laevigatum	Time	82.111	4.679	1	2506.755	25.855	1	592.649	12.571	1	295.155	8.872	1	2499.299	25.816	1
	Treatment×Time	9.809	1.617	0.986	9.935	1.628	0.987	9.426	1.585	0.983	17.084	2.134	1	157.673	6.484	1
	Treatment	1.476	0.627	0.306	38.112	3.188	1	263.558	8.383	1	45.828	3.496	1	867.366	15.208	1
P. stratiotes	Time	183.937	7.004	1	421.297	10.599	1	1051.029	16.741	1	1899.909	22.509	1	3515.153	30.617	1
	Treatment×Time	1.898	0.711	0.387	9.67	1.606	0.985	11.63	1.761	0.995	9.105	1.558	0.979	391.128	10.213	1
	Treatment	0.993	0.515	0.213	25.697	2.618	1	241.27	8.021	1	20.764	2.353	1	363.678	9.848	1
E. crassipes	Time	920.698	15.669	1	212.594	7.529	1	250.804	8.178	1	1567.135	20.443	1	3794.089	31.808	1
	Treatment×Time	5.069	1.163	0.827	3.039	0.9	0.588	35.731	3.087	1	0.96	0.506	0.207	159.824	6.528	1

Table S1. (Continued)

		POD			CAT			Root NPs			Leaves NPs			Total NPs		
		F	Effect Size	Power	F	Effect Size	Power	F	Effect Size	Power	F	Effect Size	Power	F	Effect Size	Power
L. laevigatum	Treatment	40.005	3.266	1	739.382	14.042	1	549.679	12.107	1	5907.421	39.69	1	635.456	13.017	1
	Time	312.979	9.136	1	1418.32	19.448	1	158.158	6.494	1	5857.549	39.522	1	206.85	7.427	1
	Treatment×Time	9.134	1.561	0.979	145.166	6.222	1	55.294	3.84	1	1789.049	21.842	1	59.535	3.984	1
P. stratiotes	Treatment	210.998	7.501	1	255.732	8.258	1	8509.793	47.637	1	411.879	10.48	1	5973.155	39.91	1
	Time	1392.577	19.271	1	579.383	12.43	1	1836.425	22.129	1	208.398	7.455	1	387.906	10.171	1
	Treatment×Time	48.4	3.593	1	47.562	3.561	1	600.697	12.656	1	100.632	5.18	1	1397.517	19.305	1
E. crassipes	Treatment	631.268	12.975	1	266.249	8.426	1	5581.711	38.581	1	18.397	2.215	1	2432.344	25.468	1
	Time	657.072	13.237	1	1553.89	20.356	1	5107.214	36.904	1	27.59	2.712	1	2312.196	24.831	1
	Treatment×Time	68.398	4.271	1	158.323	6.498	1	1191.967	17.829	1	4.125	1.049	0.735	457.92	11.05	1

Note: Statistical power was calculated based on the F values obtained from two-way ANOVA. Significance level $\alpha = 0.05$, with five treatments, four time, and three replicates per treatment combination ($n = 3$, total $N = 60$). Power values $\geqslant$ 0.8 indicate adequate sample size.

Table S2. Results of two-way analyses of variance.

	L. laevigatum						*P. stratiotes*						*E. crassipes*					
	Treatment		Time		Treatment×Time		Treatment		Time		Treatment×Time		Treatment		Time		Treatment×Time	
	F	*p*	F	*p*	F	*p*	F	*p*	F	*p*	F	*p*	F	*p*	F	*p*	F	*p*
Chl a	24.545	<0.001	117.008	<0.001	1.888	0.066	27.612	<0.001	72.072	<0.001	3.516	0.001	12.221	<0.001	225.252	<0.001	3.906	<0.001
Chl b	22.818	<0.001	196.752	<0.001	1.813	0.079	3.646	0.013	166.36	<0.001	8.212	<0.001	7.178	<0.001	121.029	<0.001	5.243	<0.001
Chl	32.707	<0.001	185.402	<0.001	1.679	0.109	23.287	<0.001	81.524	<0.001	2.512	0.014	13.816	<0.001	272.161	<0.001	4.607	<0.001
Root activity	91.142	<0.001	2506.755	<0.001	9.935	<0.001	38.112	<0.001	421.297	<0.001	9.67	<0.001	25.697	<0.001	212.594	<0.001	3.039	0.004
Cell membrane permeability	13.337	<0.001	41.847	<0.001	2.588	0.012	14.328	<0.001	290.164	<0.001	3.219	0.003	17.746	<0.001	160.484	<0.001	5.687	<0.001
SS	4.903	0.003	137.501	<0.001	2.525	0.014	26.239	<0.001	309.549	<0.001	7.339	<0.001	5.321	0.002	3591.013	<0.001	14.926	<0.001
SP	4.555	0.004	82.111	<0.001	9.809	<0.001	1.476	0.228	183.937	<0.001	1.898	0.065	0.993	0.423	920.698	<0.001	5.069	<0.001
Pro	180.534	<0.001	592.649	<0.001	9.426	<0.001	263.558	<0.001	1051.029	<0.001	11.63	<0.001	241.27	<0.001	250.804	<0.001	35.731	<0.001
MDA	76.104	<0.001	295.155	<0.001	17.084	<0.001	45.828	<0.001	1899.909	<0.001	9.105	<0.001	20.764	<0.001	1567.135	<0.001	0.96	0.501
SOD	507.373	<0.001	2499.299	<0.001	157.673	<0.001	867.366	<0.001	3515.153	<0.001	391.128	<0.001	363.678	<0.001	3794.089	<0.001	159.824	<0.001
POD	40.005	<0.001	312.979	<0.001	9.134	<0.001	210.998	<0.001	1392.577	<0.001	48.4	<0.001	631.268	<0.001	657.072	<0.001	68.398	<0.001
CAT	739.382	<0.001	1418.32	<0.001	145.166	<0.001	255.732	<0.001	579.383	<0.001	47.562	<0.001	266.249	<0.001	1553.89	<0.001	158.323	<0.001
Root NPs	549.679	<0.001	158.158	<0.001	55.294	<0.001	8509.793	<0.0001	1836.425	<0.001	600.697	<0.001	5581.711	<0.001	5107.214	<0.001	1191.967	<0.001
Leaves NPs	5907.421	<0.001	5857.549	<0.001	1789.049	<0.001	411.879	<0.001	208.398	<0.001	100.632	<0.001	18.397	<0.001	27.59	<0.001	4.125	0.001
Total NPs	635.456	<.001	206.85	<.001	59.535	<.001	5973.155	<.001	387.906	<.001	1397.517	<.001	2432.344	<.001	2312.196	<.001	457.92	<.001

References

[1] Wang, X. Principles and techniques of plant physiological and biochemical experiments, second ed. *Higher Education Press*: Beijing, China, **2006**.

[2] Tang, S.; Luo, C. Experimental course of plant physiology. *Southwest China Normal University Press*: Chongqing, China, **2012**.

[3] Yuan, W.; Xu, E. G.; Li, L.; Zhou, A.; Peijnenburg, W. J.G.M.; Grossart, H.; Liu, W.; Yang, Y., Tracing and trapping micro- and nanoplastics: Untapped mitigation potential of aquatic plants? *Water Res.* **2023**, *242*, 120249.
